# Curvature-dimension conditions under time change

**DOI:** 10.1007/s10231-021-01138-x

**Published:** 2021-08-04

**Authors:** Bang-Xian Han, Karl-Theodor Sturm

**Affiliations:** 1 School of Mathematical Sciences, University of Science and Technology of China, Hefei, China; 2 Institut für Angewandte Mathematik, Universität Bonn, Bonn, Germany

**Keywords:** Metric measure space, Curvature-dimension condition, Time change, Bakry–Émery theory, Dirichlet form, 53C23, 58J65, 31E05

## Abstract

We derive precise transformation formulas for synthetic lower Ricci bounds under time change. More precisely, for local Dirichlet forms we study how the curvature-dimension condition in the sense of Bakry–Émery will transform under time change. Similarly, for metric measure spaces we study how the curvature-dimension condition in the sense of Lott–Sturm–Villani will transform under time change.

## Introduction

A. Bakry and Émery [5] formulated a powerful criterion for obtaining equilibration and regularity results for the Markov semigroups associated with local Dirichlet forms. Let us briefly recall their concept. A Dirichlet form ${\mathcal {E}}$, densely defined on some $L^2(X,{\mathfrak {m}})$, satisfies the BE(*k*, *N*) condition with some function $k \in L_{\mathrm{{loc}}}^\infty (X,{\mathfrak {m}})$ and some number $N\in [2,\infty ]$ if

1 $$\begin{aligned} \frac{1}{2} \int \Gamma (f) \Delta \varphi \,{\mathrm {d}}{\mathfrak {m}}-\int \Gamma (f, \Delta f) \varphi \,{\mathrm {d}}{\mathfrak {m}}\ge \int \Big (k\, \Gamma (f)+ \frac{1}{N} (\Delta f)^2 \Big )\varphi \,{\mathrm {d}}{\mathfrak {m}}. \end{aligned}$$

for all suitable functions *f* and $\varphi \ge 0$ on *X*. Here, $\Delta$ denotes the generator associated with ${\mathcal {E}}$ and $\Gamma$ the carré du champ operator. Estimate (1) can be regarded as an abstract formulation of Bochner’s inequality on Riemannian manifolds. Thus, in this Eulerian approach to curvature-dimension conditions, *k*(*x*) will be considered as a synthetic lower bound for the “Ricci curvature at $x\in X$” and *N* as an upper bound for the “dimension” of *X*.

From the very beginning of this theory, the transformation formula for the Bakry–Émery condition ${\mathrm{{BE}}}(k,N)$ under *drift transformation* played a key role. Most importantly in the case $N=\infty$, this states that the Dirichlet form

$$\begin{aligned} {\mathcal {E}}^*(u):=\int \Gamma (u)\,{\mathrm {d}}{\mathfrak {m}}^*{\text { on }}L^2(X,{\mathfrak {m}}^*){\text { with }}{\mathfrak {m}}^*:=e^{-V}\,{\mathfrak {m}}\end{aligned}$$

satisfies ${\mathrm{{BE}}}(k^*,\infty )$ with $k^*:=k+h_V$ where $h_V(x):=\inf _f \frac{1}{\Gamma (f)}\big [\Gamma \big (\Gamma (V,f),f\big )-\frac{1}{2}\Gamma \big (\Gamma (f),V\big )\big ]$ denotes the lower bound for the Hessian of *V* at $x\in X$ for any sufficiently regular function *V* on *X*.

The goal of this paper now is to analyze the transformation property of the Bakry–Émery condition under *time change*. That is, we will pass from the original Dirichlet form ${\mathcal {E}}$ on $L^2(X,{\mathfrak {m}})$ to a new one defined as

$$\begin{aligned} {\mathcal {E}}'(u):=\int \Gamma (u)\,{\mathrm {d}}{\mathfrak {m}}{\text { on }}L^2(X,{\mathfrak {m}}'){\text { with }}{\mathfrak {m}}':=e^{2w}\,{\mathfrak {m}}\end{aligned}$$

for some $w\in L^\infty _{\mathrm{{loc}}}(X,{\mathfrak {m}})$. Our main result provides a Bakry–Émery condition for this transformed Dirichlet form provided the original Dirichlet form satisfies a Bakry–Émery condition with finite *N*. We remark that it is possible to weaken the curvature-dimension condition adopted in this theorem to a milder distributional condition, in the spirit of the papers [11, 27].

### **Theorem 1**

*Assume that* ${\mathcal {E}}$ *satisfies the* ${\text {BE}}(K, \infty )$ *condition for some* $K\in {\mathbb {R}}$ *and the* ${\text {BE}}(k,N)$ *condition for some* $k \in L^\infty _{\mathrm{{loc}}}$ *and some* $N\in [1,\infty )$, *and that* $w\in {D}_{\mathrm{{loc}}}({ \varvec{\Delta }}) \cap L_{\mathrm{{loc}}}^\infty$ *with* ${\varvec{\Delta }} w={\varvec{\Delta }}_\mathrm{{sing}} w+{ \Delta }_\mathrm{{ac}} w \, {\mathfrak {m}}$ *and* ${\varvec{\Delta }}_\mathrm{{sing}} w \le 0$. *Then, for any* $N'\in (N, \infty ]$ and $k' \in L_{\mathrm{{loc}}}^\infty$, *the time-changed Dirichlet form* ${\mathcal {E}}'$ on $L^2(X,{\mathfrak {m}}')$ *satisfies the* ${\text {BE}}(k', N')$ *condition provided*

2 $$\begin{aligned} k'\le e^{-2w}\Big [{k}- \frac{(N-2)(N'-2)}{N'-N} \Gamma (w)- {\Delta }_\mathrm{{ac}} w \Big ] \end{aligned}$$

$${\mathfrak {m}}$$-a.e. on *X*.

### **Corollary 1**

If in addition $k'$ is bounded from below, say $k'\ge K'$ for some $K'\in {\mathbb {R}}$, then the time-changed Dirichlet form ${\mathcal {E}}'$ and the associated heat semigroup $(P'_t)_{t\ge 0}$ satisfy the following gradient estimate

3 $$\begin{aligned} \Gamma '(P'_t f) +\frac{1-e^{-2K't}}{N'K'} (\Delta ' P'_t f)^2\le e^{-2K't} P'_t \big (\Gamma '(f)\big ). \end{aligned}$$

### *Remark 1*

Generator and carré du champ operator of the time-changed Dirichlet form ${\mathcal {E}}'$ on $L^2(X,{\mathfrak {m}}')$ are given by

$$\begin{aligned} \Delta '=e^{-2w} \Delta , ~~ \Gamma '=e^{-2w} \Gamma . \end{aligned}$$

Moreover, the associated Brownian motion $({\mathbb {P}}'_x, B'_t)$ (cf. Chapter 6 [12]) is given by ${\mathbb {P}}'_x={\mathbb {P}}_x$ and

4 $$\begin{aligned} B'_t=B_{\tau _t},\quad \tau _t=\int _0^t e^{-2w(B'_s)}{\mathrm {d}}s,\qquad \sigma _t=\int _0^t e^{2w (B_s)}\, {\mathrm {d}}s,\quad B_t=B'_{\sigma _t}. \end{aligned}$$

Note that heat semigroup $(P'_t)_{t\ge 0}$ and Brownian motion $({\mathbb {P}}'_x, B'_t)$ are linked to each other by

$$\begin{aligned} P'_t f(x) ={\mathbb {E}}'_x[f(B'_{2t})]. \end{aligned}$$

B. A different approach, the so-called Lagrangian approach, to synthetic lower Ricci bounds was proposed in the works of Lott and Villani [19] and Sturm [23]. Here, the objects under consideration are metric measure spaces. Such a space $(X,{\mathrm {d}},{\mathfrak {m}})$ satisfies the curvature-dimension condition CD$(K,\infty )$—meaning that its Ricci curvature is bounded from below by *K*—if the Boltzmann entropy ${\text {Ent}}(.,{\mathfrak {m}})$ is weakly *K*-convex on the Wasserstein space ${{\mathcal {P}}}_2(X)$. More refined curvature-dimension conditions CD(*K*, *N*) and ${\text {CD}}^*(K,N)$ with finite $N\in [1,\infty )$ were introduced in [23, 24]. Combined with the requirement of Hilbertian energy functional, this led to the conditions RCD(*K*, *N*) and ${\text {RCD}}^*(K,N)$ [2], which fortunately turned out to be equivalent to each other [7].

Also from the very beginning of this theory, the transformation formula for the curvature-dimension conditions CD(*K*, *N*), ${\text {CD}}^*(K,N)$ RCD(*K*, *N*) under *drift transformation* played a key role. Most easily formulated in the case $N=\infty$, it states that the condition CD$(K,\infty )$ for a given metric measure space $(X,{\mathrm {d}},{\mathfrak {m}})$ and the *L*-convexity of *V* on *X* imply the condition CD$(K+L,\infty )$ for the transformed metric measure space $(X,{\mathrm {d}},e^{-V}{\mathfrak {m}})$. The same holds with RCD in the place of CD.

Subject of the investigations in this paper is the *time-changed metric measure space* $(X,{\mathrm {d}}',{\mathfrak {m}}')$ where ${\mathfrak {m}}'=e^{2w}{\mathfrak {m}}$ for some $w\in L^\infty _{\mathrm{{loc}}}(X,{\mathfrak {m}})$ and

$$\begin{aligned} {\mathrm {d}}'(x, y):=\sup \big \{\phi (x)-\phi (y): \phi \in {D}_{\mathrm{{loc}}}({\mathcal {E}}) \cap C(X), |\mathrm {D}\phi | \le e^w \ {\mathfrak {m}}{\text {-a.e. in}}~ X \big \} \end{aligned}$$

for $x, y \in X$. Assuming that *w* is continuous ${\mathfrak {m}}$-a.e. on *X* this allows for a dual representation as

$$\begin{aligned} {\mathrm {d}}'(x, y)=\inf \Big \{\int _0^1 e^{{\bar{w}} (\gamma _s)}\, |{\dot{\gamma }}_s|\,{\mathrm {d}}s: \gamma \in \hbox {AC}([0,1], X), \gamma _0=x,\gamma _1=y\Big \} \end{aligned}$$

where ${\bar{w}}(x):=\limsup _{y\rightarrow x}w(y)$ denotes the upper semicontinuous envelope of *w*. Our main result provides the transformation formula for the curvature-dimension condition under time change.

### **Theorem 2**

*Let* $(X,{\mathrm {d}},{\mathfrak {m}})$ *be an* ${\mathrm{{RCD}}}(K, N)$ *space for some* $K\in {\mathbb {R}}$ *and* $N\in [1, \infty )$, *and let* $w\in {D}_{\mathrm{{loc}}}({\varvec{\Delta }})\cap L^\infty _{\mathrm{{loc}}}(X)$ *be continuous* ${\mathfrak {m}}$*-a.e. with* ${\varvec{\Delta }} w={\varvec{\Delta }}_\mathrm{{sing}} w+{ \Delta }_\mathrm{{ac}} w \, {\mathfrak {m}}$ *and* ${\varvec{\Delta }}_\mathrm{{sing}} w \le 0$. *Then, the time-changed metric measure space* $(X, {\mathrm {d}}', {\mathfrak {m}}')$ *satisfies the* $\mathrm{RCD}(K', N')$ *condition for any* $N'\in (N, \infty )$ *and* $K'\in {\mathbb {R}}$ *such that*

$$\begin{aligned} K'\le e^{-2w}\Big [K- \frac{(N-2)(N'-2)}{N'-N} |\mathrm {D}w|^2- { \Delta }_\mathrm{{ac}} w \Big ]. \end{aligned}$$

Theorem 2 is a more or less immediate consequence of Theorem 1 and the fact that the Eulerian and the Lagrangian curvature-dimension conditions, BE(*K*, *N*) and RCD(*K*, *N*), are equivalent to each other as proven in [10].

### *Remark 2*

The first derivation of the transformation formula for the (Eulerian) curvature-dimension condition BE(*K*, *N*) under conformal transformation as well as under time change was presented in [26] by the second author in the setting of regular Dirichlet forms admitting a nice core of sufficiently smooth functions (“$\Gamma$-calculus in the sense of Bakry–Émery–Ledoux”).

Combining the techniques and results in [14, 20], the first author [15, 16] proved the transformation formula for the Lagrangian curvature-dimension condition ${\mathrm{{RCD}}}(K, N)$ under conformal transformation when the reference function *w* is bounded and smooth enough. Together with the well-known transformation formula for ${\mathrm{{RCD}}}(K, N)$ under drift transformations, this result also provides a transformation formula for ${\mathrm{{RCD}}}(K, N)$ under time change.

The focus of the current paper is on proving the transformation formula for the (Eulerian or Lagrangian) curvature-dimension condition under time change in a setting of great generality (Dirichlet forms or metric measure spaces) and with minimal regularity and boundedness assumptions on *w*.

C. One of the important applications of time-change is the “convexification” of non-convex subsets $\Omega \subset X$ of an RCD(*K*, *N*)-space $(X,{\mathrm {d}},{\mathfrak {m}})$ as introduced by the second author and Lierl [18]. For sublevel sets of regular semi-convex functions *V*, they proved convexity after suitable conformal transformations, while control of the curvature bound under these transformations follows from the work [15] of the first author. Unfortunately, these previous results do not apply to the most natural potential, the signed distance function $V={\mathrm {d}}(.,\Omega )-{\mathrm {d}}(.,X{\setminus }\Omega )$ due to lack of regularity. The more general results of the current paper will apply to a suitable truncation of the signed distance function and thus provide the following convexification theorem.

### **Theorem 3**

*Let* $(X,{\mathrm {d}},{\mathfrak {m}})$ *be an* ${\mathrm{{RCD}}}(K, N)$ *space and* $\Omega$ *be a bounded* $\ell$*-convex domain in* $(X, {\mathrm {d}})$ *with* ${\mathfrak {m}}(\partial \Omega )=0$ *and* ${\mathfrak {m}}^+(\partial \Omega )<\infty$. *Then, for any* $N'\in (N, +\infty ]$*, there exists a Lipschitz function* *w* *such that the time-changed metric measure space* $({\overline{\Omega }}, {\mathrm {d}}^{w}, {\mathfrak {m}}^w)$ *is a* $\mathrm{RCD}(K', N')$ *space for some* $K' \in {\mathbb {R}}$.

## Time change and the Bakry–Émery condition

This section is devoted to study synthetic lower Ricci bounds under time change in the setting of Dirichlet forms. More precisely, we will derive the transformation formula for the Bakry–Émery condition under time change.

### Dirichlet forms and the ${\mathrm{{BE}}}(K, N)$ condition

In this part, we recall some basic facts about Dirichlet form theory and the Bakry–Émery theory. Firstly we make some basic assumptions on the Dirichlet form, see also [21] for examples satisfying these conditions.

#### **Assumption 1**

We assume that

- $(X, \tau )$ is a topological space, $(X, {\mathcal {B}})$ is a measurable space and ${\mathfrak {m}}$ is a $\sigma$-finite Radon measure with full support (i.e., $\mathop {\mathrm{{supp}}}\nolimits {\mathfrak {m}}=X$); ${{\mathcal {B}}}$ is the ${\mathfrak {m}}$-completion of the Borel $\sigma$-algebra generated by $\tau$; and $L^p(X, {\mathfrak {m}})$ will denote the space of $L^p$-integrable functions on $(X, {\mathcal {B}}, {\mathfrak {m}})$;
- ${\mathcal {E}}(\cdot ): L^2(X, {\mathfrak {m}}) \rightarrow [0,\infty ]$ is a strongly local, quasi-regular, symmetric Dirichlet form with domain ${{\mathbb {V}}}:={D}({\mathcal {E}})=\big \{ f\in L^2(X, {\mathfrak {m}}): {\mathcal {E}}(f) < \infty \big \}$; denote by $({P}_t)_{t>0}$ the heat semi-group generated by ${\mathcal {E}}$;
- there exists an increasing sequence of (“cut-off”) functions with compact support $({{\chi }}_\ell )_{\ell \ge 1}\subset {\mathbb {V}}$ such that $0\le {{\chi }}_\ell \le 1$, $\Gamma ({{\chi }}_\ell )\le C$ for all $\ell$ and ${{\chi }}_\ell \rightarrow 1$, $\Gamma ({{\chi }}_\ell ) \rightarrow 0$ ${\mathfrak {m}}$-a.e. as $\ell \rightarrow \infty$, cf. [22];
- ${\mathcal {E}}$ satisfies the Bakry–Émery condition ${\mathrm{{BE}}}(K, \infty )$ for some $K\in {\mathbb {R}}$.

To formulate the latter, recall that ${\mathbb {V}}_\infty :={D}({\mathcal {E}}) \cap L^\infty (X, {\mathfrak {m}})$ is an algebra with respect to pointwise multiplication. We say that ${\mathcal {E}}$ admits a carré du champ if there exists a quadratic continuous map $\Gamma : {\mathbb {V}}\rightarrow L^1(X, {\mathfrak {m}})$ such that

$$\begin{aligned} \int _X\Gamma (f) \varphi \,{\mathrm {d}}{\mathfrak {m}}={\mathcal {E}}(f, f\varphi )-\frac{1}{2}{\mathcal {E}}(f^2,\varphi )\qquad {\text {for all }}f\in {\mathbb {V}},\varphi \in {\mathbb {V}}_\infty . \end{aligned}$$

By polarization, we define $\Gamma (f, g) :=\frac{1}{4} \big ( \Gamma (f+g)-\Gamma (f-g)\big )$ and obtain ${\mathcal {E}}(f,g)=\int \Gamma (f,g)\,{\mathrm {d}}{\mathfrak {m}}$ for all $f, g\in {\mathbb {V}}$. It is known that $\Gamma$ is local in the sense that $\Gamma (f-g)=0$ ${\mathfrak {m}}$-a.e. on the set $\{f=g\}$.

The Dirichlet form ${\mathcal {E}}$ induces a densely defined self-adjoint operator $\Delta :{D}(\Delta )\subset {\mathbb {V}}\rightarrow L^2$ satisfying ${\mathcal {E}}(f,g)=-\int g\Delta f\,{\mathrm {d}}{\mathfrak {m}}$ for all $g\in {\mathbb {V}}$. Put

$$\begin{aligned} \Gamma _2(f; \varphi ):=\frac{1}{2} \int \Gamma (f) \Delta \varphi \,{\mathrm {d}}{\mathfrak {m}}-\int \Gamma (f, \Delta f) \varphi \,{\mathrm {d}}{\mathfrak {m}}\end{aligned}$$

and $${D}(\Gamma _2):=\Big \{ (f, \varphi ): f,\varphi \in \mathrm{D}(\Delta ), \ \Delta f\in {\mathbb {V}}, \ \varphi ,\Delta \varphi \in {L^\infty }\Big \}$$.

#### **Definition 1**

(Bakry–Émery condition) Given a function ${k} \in L^\infty$ and a number $N\in [1,\infty ]$, we say that the Dirichlet form ${\mathcal {E}}$ satisfies the BE(*k*, *N*) condition if it admits a carré du champ and if

5 $$\begin{aligned} \frac{1}{2} \int \Gamma (f) \Delta \varphi \,{\mathrm {d}}{\mathfrak {m}}-\int \Gamma (f, \Delta f) \varphi \,{\mathrm {d}}{\mathfrak {m}}\ge \int \Big ({k} \Gamma (f)+ \frac{1}{N} (\Delta f)^2 \Big )\varphi \,{\mathrm {d}}{\mathfrak {m}}. \end{aligned}$$

for all $$(f, \varphi )\in {D}(\Gamma _2)$$, $$\varphi \ge 0$$.

#### *Remark 3*

Since by our standing assumption the Dirichlet form ${\mathcal {E}}$ satisfies ${\mathrm{{BE}}}(K, \infty )$ for some $K\in {\mathbb {R}}$, the “space of test functions”

$$\begin{aligned} \mathrm{TestF}({\mathcal {E}}):= \big \{f\in {D}(\Delta ): \ \Delta f\in {\mathbb {V}}^\infty , \, \Gamma (f)\in L^\infty \big \} \end{aligned}$$

is dense in ${\mathbb {V}}$ (cf. Section 2 [3] and Remark 2.5 therein). Hence, the BE(*k*, *N*) condition will follow if (5) holds true for all $f\in \mathrm{TestF}({\mathcal {E}})$ and all non-negative $\varphi \in {D}(\Delta )\cap L^\infty$ with $\Delta \varphi \in {L^\infty }$.

#### **Lemma 1**

*For every* $f\in {D}(\Delta )$, *we have* $\Gamma (f)^{1/2}\in {\mathbb {V}}$ *and*

$$\begin{aligned} {\mathcal {E}}\Big (\Gamma (f)^{1/2}\Big )\le \int (\Delta f)^2\,{\mathrm {d}}{\mathfrak {m}}- K\cdot {\mathcal {E}}(f). \end{aligned}$$

#### *Proof*

By self-improvement, the Bakry–Émery inequality BE$(K,\infty )$ as introduced above implies the stronger $L^1$-version

$$\begin{aligned} \int \Gamma (f)^{1/2} \Delta \varphi \,{\mathrm {d}}{\mathfrak {m}}- \int \frac{1}{\Gamma (f)^{1/2}} \Gamma (f, \Delta f) \varphi \,{\mathrm {d}}{\mathfrak {m}}\ge K\,\int \Gamma (f)^{1/2}\varphi \,{\mathrm {d}}{\mathfrak {m}}. \end{aligned}$$

for all $f,\varphi \in {D}(\Delta )$ with $\Delta f\in {\mathbb {V}}$, see [20]. Choosing $\varphi =P_t(\Gamma (f)^{1/2})$ and then letting $t\rightarrow 0$ yields the claim for $f\in {D}(\Delta )$ with $\Delta f\in {\mathbb {V}}$. Since the class of these *f*’s is dense in $\mathrm{D}(\Delta )$, the claim follows. $\square$

#### **Definition 2**

(i) We say that $f \in {\mathbb {V}}^e$ if there exists a Cauchy sequence $( f_n )_n \subset {\mathbb {V}}$ w.r.t. the semi-norm ${\mathcal {E}}( \cdot )$ and such that $f_n \rightarrow f$ ${\mathfrak {m}}$-a.e. Then, we define ${\mathcal {E}}(f):=\mathop {\lim }_{{n} \rightarrow {\infty }} {\mathcal {E}}(f_n)$. Similarly, $\Gamma$ can be extended to ${\mathbb {V}}^e$.

(ii) We say that $f\in {\mathbb {V}}_{\mathrm{{loc}}}$ if for any bounded open set *U*, there is ${\bar{f}}\in {\mathbb {V}}$ such that $f={\bar{f}}$ on *U*. Then, a function $\Gamma (f)\in L^1_{\mathrm{{loc}}}(X, {\mathfrak {m}})$ can be defined unambiguously by $\Gamma (f):=\Gamma ({\bar{f}})$ on *U*.

Similarly, we define the spaces ${D}_{\mathrm{{loc}}}(\Delta )$ and $\mathrm{TestF}_{\mathrm{{loc}}}({\mathcal {E}})$.

#### **Definition 3**

(*Local weak Bakry–Émery condition*) Given a function ${k} \in L^\infty _{\mathrm{{loc}}}$ and a number $N\in [1,\infty ]$, we say that the Dirichlet form ${\mathcal {E}}$ satisfies the ${\text {BE}}_{\mathrm{{loc}}}({k},N)$ condition if it admits a carré du champ and if

6 $$\begin{aligned} -\frac{1}{2} \int \Gamma \big (\Gamma (f), \varphi \big )\,{\mathrm {d}}{\mathfrak {m}}-\int \Gamma (f, \Delta f) \varphi \,{\mathrm {d}}{\mathfrak {m}}\ge \int \Big ({k} \Gamma (f)+ \frac{1}{N} (\Delta f)^2 \Big )\varphi \,{\mathrm {d}}{\mathfrak {m}}. \end{aligned}$$

for all $f\in {D}_{\mathrm{{loc}}}(\Delta )\cap L^\infty _{\mathrm{{loc}}}$ with $\Delta f\in {\mathbb {V}}_{\mathrm{{loc}}}$ and all non-negative $\varphi \in {\mathbb {V}}^\infty$ with compact support and $\Gamma (\varphi )\in L^\infty$.

Note that our standing assumption ${\mathrm{{BE}}}(K, \infty )$ implies that $\Gamma (f)^{1/2}\in {\mathbb {V}}_{\mathrm{{loc}}}$ for each $f\in {D}_{\mathrm{{loc}}}(\Delta )$. Thus, for functions *f* and $\varphi$ as above, the term $-\frac{1}{2} \int \Gamma \big (\Gamma (f),\varphi \big )\,{\mathrm {d}}{\mathfrak {m}}$ is well defined.

#### **Lemma 2**

${\mathcal {E}}$ *satisfies* ${\text {BE}}(k,N)$ *for* ${k} \in L^\infty$ *if and only if it satisfies* ${\text {BE}}_{\mathrm{{loc}}}(k,N)$.

#### *Proof*

Assume that BE(*k*, *N*) holds true and let *f* and $\varphi$ be given as in Definition 3. Choose $f'\in \mathrm{D}(\Delta )\cap L^\infty$ with $\Delta f'\in {\mathbb {V}}$ such that $f=f'$ on a neighborhood of $\{\varphi \not =0\}$. Choose uniformly bounded, nonnegative $\varphi _n\in {D}(\Delta )$ with $\Gamma (\varphi _n), \Delta \varphi _n \in {L^\infty }$ such that $\varphi _n\rightarrow \varphi$ a.e. on X and in ${\mathbb {V}}$ as $n\rightarrow \infty$. (For instance, put $\varphi _n=P_{1/n}\varphi$.) Then, (5) implies

$$\begin{aligned} -\frac{1}{2} \int \Gamma \big (\Gamma (f'), \varphi _n\big )\,{\mathrm {d}}{\mathfrak {m}}-\int \Gamma (f', \Delta f') \varphi _n\,{\mathrm {d}}{\mathfrak {m}}\ge \int \Big ({k} \Gamma (f')+ \frac{1}{N} (\Delta f')^2 \Big )\varphi _n\,{\mathrm {d}}{\mathfrak {m}}>-\infty \end{aligned}$$

for all *n*. Passing to the limit $n\rightarrow \infty$ yields (6) with $f'$ in the place of *f*. Since by assumption $f=f'$ on a neighborhood of $\{\varphi \not =0\}$, this yields the claim (6).

Conversely, assume that ${\hbox {BE}}_{\mathrm{{loc}}}({k},N)$ holds true and let *f* and $\varphi$ be given as in Definition 1. Put $\varphi _n= P_{1/n}\varphi$ and $\varphi _{\ell ,n}={{\chi }}_\ell \cdot P_{1/n}\varphi$ with $({{\chi }}_\ell )_\ell$ being the cut-off functions from assumption 1. According to the ${\hbox {BE}}_{\mathrm{{loc}}}({k},N)$ assumption, (6) holds with $\varphi _{\ell ,n}$ in the place of $\varphi$. Passing to the limit $\ell \rightarrow \infty$ yields (6) with $\varphi _{n}$ in the place of $\varphi$ ($\forall n$). This, however, is equivalent to (5), again with $\varphi _{n}$ in the place of $\varphi$. Finally passing to the limit $n\rightarrow \infty$ yields (5) for the given $\varphi$. $\square$

#### *Remark 4*

From the proof of the preceding lemma, it is obvious that the class of *f*’s to be considered for (6) can equivalently be restricted to $f\in {D}_{\mathrm{{loc}}}(\Delta )\cap L^\infty _{\mathrm{{loc}}}$ with $\Delta f\in {\mathbb {V}}_{\mathrm{{loc}}}\cap L^\infty _{\mathrm{{loc}}}$.

### Self-improvement of the Bakry–Émery condition

The formulation of the subsequent results on the self-improvement property will require the theory of differential structures of Dirichlet forms as introduced by Gigli [14]. In order to shorten the length of the paper, we will skip the introduction of (co)tangent modules, list the results directly and ignore subtle differences.

#### **Proposition 1**

(Section 2.2, [14]) *Given a strongly local, symmetric Dirichlet form* ${\mathcal {E}}$ *admitting a carré du champ* $\Gamma$ *defined on* ${\mathbb {V}}^e$ *as above. Then, there exists an* $L^\infty$*-Hilbert module* $L^2(TX)$ *satisfying the following properties.*

(i) $L^2(TX)$ *is a Hilbert space equipped with the norm* $\Vert \cdot \Vert$ *such that the following correspondence (embedding) holds*
  $$\begin{aligned} {\mathbb {V}}^e \ni f \rightarrow \nabla f \in L^2(TX), ~~~~\Vert \nabla f\Vert ^2=\int \Gamma (f)\,{\mathrm {d}}{\mathfrak {m}}. \end{aligned}$$
(ii) $L^2(TX)$ *is a module over the commutative ring* $L^\infty (X, {\mathfrak {m}})$.
(iii) *The norm* $\Vert \cdot \Vert$ *is induced by a pointwise inner product* ${\langle }\cdot , \cdot {\rangle }$ *satisfying*
  $$\begin{aligned} {\langle }\nabla f, \nabla g {\rangle }=\Gamma (f,g)~~~~~{\mathfrak {m}}{\text {-a.e.}} \end{aligned}$$
  and
  $$\begin{aligned} {\langle }h \nabla f, \nabla g {\rangle }=h{\langle }\nabla f, \nabla g {\rangle }~~~~~{\mathfrak {m}}{\text {-a.e.}} \end{aligned}$$
  *for any* $f, g\in {\mathbb {V}}^e$ *and* $h\in L^\infty (X, {\mathfrak {m}})$.
(iv) $L^2(TX)$ *is generated by* $\{\nabla g: g\in {\mathbb {V}}^e\}$ *in the following sense. For any* $v\in L^2(TX)$, *there exists a sequence* $v_n=\sum _{i=1}^{M_n} a_{n,i} \nabla g_{n,i}$ *with* $a_{n,i}\in L^\infty$ *and* $g_{n,i}\in {\mathbb {V}}^e$, *such that* $\Vert v-v_n\Vert \rightarrow 0$ *as* $n\rightarrow \infty$.

By Corollary 3.3.9 [14], for any $f \in {D}({\Delta })$ there is a continuous symmetric $L^\infty (M)$-bilinear map ${\mathrm {Hess}}_f(\cdot , \cdot )$ defined on $[L^2(TX)]^2$, with values in $L^0(X, {\mathfrak {m}})$. In particular, if $f, g, h \in \mathrm{TestF}({\mathcal {E}})$ (cf. Lemma 3.2 [20], Theorem 3.3.8 [14]), ${\mathrm {Hess}}_f(\cdot , \cdot )$ is given by the following formula:

7 $$\begin{aligned} 2{\mathrm {Hess}}_f(\nabla g, \nabla h)=\Gamma (g, \Gamma (f, h)) +\Gamma (h, \Gamma (f, g))-\Gamma (f, \Gamma (g,h)). \end{aligned}$$

Combining Theorem 1.4.11 and Proposition 1.4.10 in [14], we obtain the following structural results. As a consequence, we can compute ${\mathrm {Hess}}_f(\cdot , \cdot )$ and $\Gamma (\cdot , \cdot )$ using local coordinates.

#### **Proposition 2**

*Denote by* $L^2(TX)$ *the tangent module associated with* ${\mathcal {E}}$. *Then, there exists a unique decomposition (up to* ${\mathfrak {m}}$*-null sets)* $\{ E_n\}_{n \in {\mathbb {N}} \cup \{\infty \}}$ *of* *X* *such that*

- *For any* $n \in {\mathbb {N}}$ *and any* $B \subset E_n$ *with positive measure,* $L^2(TX)$ *has an orthonormal basis* $\{e_{i,n}\}_{i=1}^n$ *on* *B*,
- *For every subset* *B* of $E_\infty$ *with finite positive measure, there exists an orthonormal basis* $\{e_{i,B}\}_{i \in {\mathbb {N}} \cup \{\infty \}} \subset L^2(TX){|_{B}}$ *which generates* $L^2(TX){|_{B}}$,

*where we say that a countable set* $\{v_i\}_i\subset L^2(TX)$ *is orthonormal on B if* ${\langle }v_i, v_j {\rangle }=\delta _{ij}$ ${\mathfrak {m}}$*-a.e. on* *B*. *By definition, the local dimension* $\dim _{\mathrm{loc}}(x) \in {\mathbb {N}} \cup \{\infty \}$ *is* *n* *if* $x\in E_n$.

#### **Proposition 3**

*Let* ${\mathcal {E}}$ *be a Dirichlet form satisfying the* ${\text {BE}}({k},N)$ *condition for some* ${k} \in L^\infty$ *and some number* $N\in [1,\infty ]$ *and let* $\{ E_n\}_{n \in {\mathbb {N}} \cup \{\infty \}}$ *be the decomposition given by Proposition* 2. *Then,* ${\mathfrak {m}}(E_n)=0$ *for* $n>N$, *and for any* $(f, \varphi ) \in \mathrm{D}(\Gamma _2)$, *we have*

8 $$\begin{aligned} { \Gamma }_2(f; \varphi )\ge & {} \int \Big {(} {k}\Gamma (f)+|{\mathrm {Hess}}_f|^2_{\mathrm{HS}}+\frac{1}{N-\dim _{\mathrm{loc}}}({\mathrm{{tr}}}{\mathrm {Hess}}_f -\Delta f)^2 \Big {)}\varphi \,{\mathrm {d}}{\mathfrak {m}}\end{aligned}$$

*where* $\frac{1}{N-\dim _{\mathrm{loc}}}({\mathrm{{tr}}}{\mathrm {Hess}}_f -\Delta f)^2$ *is taken* 0 on $E_N$ *by definition.*

*The same estimate* (8) *also holds true for all* $f\in {D}_{\mathrm{{loc}}}(\Delta )\cap L^\infty _{\mathrm{{loc}}}$ with $\Delta f\in {\mathbb {V}}_{\mathrm{{loc}}}$ *and all non-negative* $\varphi \in {\mathbb {V}}^\infty$ *with compact support and* $\Gamma (\varphi )\in L^\infty$ *provided* ${\mathcal {E}}$ *satisfies the* ${\text {BE}}_{\mathrm{{loc}}}({k},N)$ *condition for some* ${k} \in L^\infty _{\mathrm{{loc}}}$ *and* $N\in [1,\infty ]$.

#### *Proof*

The proof for constant ${k}=K$ was given in [16], Proposition 3.2 and Theorem 3.3. In fact, the proof there only relies on a so-called self-improvement technique in Bakry–Émery theory, which can also be applied to BE(*k*, *N*) case without difficulty. Also the extension via localization is straightforward. $\square$

In order to proceed, we briefly recall the notion of measure-valued Laplacian ${\varvec{\Delta }}$ as introduced in [13, 20]. We say that $f\in {D}({\varvec{\Delta }}) \subset {\mathbb {V}}^e$ if there exists a signed Borel measure $\mu =\mu _+-\mu _-$ charging no capacity zero sets such that

$$\begin{aligned} \int \overline{\varphi } \,{\mathrm {d}}\mu =-\int \Gamma (\varphi , f)\,{\mathrm {d}}{\mathfrak {m}}\end{aligned}$$

for any $\varphi \in {\mathbb {V}}$ with quasi-continuous representative $\overline{\varphi }\in L^1(X, |\mu |)$. If $\mu$ is unique, we denote it by ${\varvec{\Delta }} f$. If ${\varvec{\Delta }} f \ll {\mathfrak {m}}$, we also denote its density by $\Delta f$ if there is no ambiguity.

#### **Proposition 4**

(See Lemma 3.2 [20]) *Let* ${\mathcal {E}}$ *be a Dirichlet form satisfying the* ${\text {BE}}_{\mathrm{{loc}}}({k},N)$ *condition. Then, for any* $f\in \mathrm{TestF}_{\mathrm{{loc}}}({\mathcal {E}})$, *we have* $\Gamma (f) \in {D}_{\mathrm{{loc}}}({\varvec{\Delta }})$ *and*

$$\begin{aligned} \frac{1}{2} {\varvec{\Delta }} \Gamma (f)-\Gamma (f, \Delta f)\,{\mathfrak {m}}\ge \Big {(} {k}\Gamma (f)+|{\mathrm {Hess}}_f|^2_{\mathrm{HS}}+\frac{1}{N-\dim _\mathrm{loc}}({\mathrm{{tr}}}{\mathrm {Hess}}_f -\Delta f)^2 \Big {)}\,{\mathfrak {m}}. \end{aligned}$$

*In particular, the singular part of the measure* ${\varvec{\Delta }} \Gamma (f)$ *is non-negative.*

### ${\mathrm{{BE}}}(K, N)$ condition under time change

We define the time change of the Dirichlet form ${\mathcal {E}}$ in the following way.

#### **Definition 4**

(*Time change*) Given a function $w\in L^\infty _{\mathrm{{loc}}}(X, {\mathfrak {m}})$, define the weighted measure ${\mathfrak {m}}^w:=e^{2w}{\mathfrak {m}}$ and the time-changed Dirichlet form ${\mathcal {E}}^w$ on $L^2(X, {\mathfrak {m}}^w)$ by

$$\begin{aligned} {\mathcal {E}}^w(f):=\int \Gamma (f)\,{\mathrm {d}}{\mathfrak {m}}~~~~~\forall f\in {\mathbb {V}}^w \end{aligned}$$

with ${D}({\mathcal {E}}^w):={\mathbb {V}}^w:={\mathbb {V}}^e \cap L^2(X, {\mathfrak {m}}^w)$. Note that indeed ${\mathcal {E}}^w(f)$ does not depend on *w* and $({\mathbb {V}}^w)^e={\mathbb {V}}^e$.

We leave it to the reader to verify the following simple but fundamental properties.

#### **Lemma 3**

(i) ${\mathcal {E}}^w$ *is a strongly local, symmetric Dirichlet form.*
(ii) ${\mathcal {E}}^w$ *admits a carré du champ defined on* $({\mathbb {V}}^w)_{\mathrm{{loc}}}={\mathbb {V}}_{\mathrm{{loc}}}$ *by* $\Gamma ^w:=e^{-2w}\Gamma$.
(iii) *Furthermore,* ${D}_{\mathrm{{loc}}}(\Delta ^w)={D}_{\mathrm{{loc}}}(\Delta )$ *and* $\Delta ^w f=e^{-2w}\Delta f$.
(iv) *If in addition* $w\in {\mathbb {V}}_{\mathrm{{loc}}}$ *then* $\mathrm{TestF}_{\mathrm{{loc}}}({\mathcal {E}}^w)=\mathrm{TestF}_{\mathrm{{loc}}}({\mathcal {E}})$.

Our first main result will provide the basic estimate for the Bakry–Émery condition under time change.

#### **Theorem 4**

*Let* $w\in {D}_{\mathrm{{loc}}}({\varvec{\Delta }}) \cap L^\infty _{\mathrm{{loc}}}$ *be given and assume that* ${\mathcal {E}}$ *satisfies* ${\text {BE}}_{\mathrm{{loc}}}({k}, N)$ *condition for some* $N\in [2, \infty )$ and ${k} \in L^\infty _{\mathrm{{loc}}}$. *Then, for any* $N'\in (N, \infty ]$, *any* $f\in \mathrm{TestF}_{\mathrm{{loc}}}({\mathcal {E}})$ *and any non-negative* $\varphi \in {\mathbb {V}}_\infty$ *with compact support, we have*

9 $$\begin{aligned}&- \int \Big [\frac{1}{2}\Gamma ^w\big (\Gamma ^w(f), \varphi \big )+\Gamma ^w\big (f, \Delta ^w f \big )\varphi \Big ]\,{\mathrm {d}}{\mathfrak {m}}^w\nonumber \\&\quad \ge \int \overline{\Gamma ^w(f)\varphi }\,{\mathrm {d}}\kappa +\frac{1}{N'}\int (\Delta ^w f)^2\varphi \,{\mathrm {d}}{\mathfrak {m}}^w \end{aligned}$$

*where*

$$\begin{aligned} \kappa := e^{-2w}\left( {k}- \frac{(N-2)(N'-2)}{N'-N} \Gamma (w)\right) {\mathfrak {m}}^w- {\varvec{\Delta }} w. \end{aligned}$$

#### *Proof*

By Lemma 3 we know

$$\begin{aligned}&-\frac{1}{2} \int \Gamma ^w\big (\Gamma ^w(f), \varphi \big )\,{\mathrm {d}}{\mathfrak {m}}^w-\int \Gamma ^w(f, \Delta ^w f) \varphi \,{\mathrm {d}}{\mathfrak {m}}^w\\&\quad = -\frac{1}{2} \int \Gamma \big ( e^{-2w}\Gamma (f), \varphi \big )\,{\mathrm {d}}{\mathfrak {m}}-\int \Gamma (f, e^{-2w}\Delta f) \varphi \,{\mathrm {d}}{\mathfrak {m}}\\&\quad = \Big (-\frac{1}{2} \int \Gamma \big ( \Gamma (f), \varphi \big )e^{-2w}\,{\mathrm {d}}{\mathfrak {m}}+\int \Gamma (f)\Gamma (w,\varphi )e^{-2w}\,{\mathrm {d}}{\mathfrak {m}}\Big )-\Big (\int \Gamma (f, \Delta f)e^{-2w} \varphi \,{\mathrm {d}}{\mathfrak {m}}\\&\qquad -2\int \Delta f\Gamma (f, w) e^{-2w} \varphi \,{\mathrm {d}}{\mathfrak {m}}\Big )\\&\quad = \Big ( -\frac{1}{2} \int \Gamma \big ( \Gamma (f), e^{-2w}\varphi \big )\,{\mathrm {d}}{\mathfrak {m}}- \int \Gamma \big ( \Gamma (f), w\big )e^{-2w} \varphi \,{\mathrm {d}}{\mathfrak {m}}\Big )+\int \Gamma (f)\Gamma (w,\varphi )e^{-2w}\,{\mathrm {d}}{\mathfrak {m}}\\&\qquad -\int \Gamma (f, \Delta f)e^{-2w} \varphi \,{\mathrm {d}}{\mathfrak {m}}+2\int \Delta f\Gamma (f, w) e^{-2w} \varphi \,{\mathrm {d}}{\mathfrak {m}}\\&\quad = \underbrace{ \Big \{ -\frac{1}{2} \int \Gamma \big ( \Gamma (f), e^{-2w}\varphi \big )\,{\mathrm {d}}{\mathfrak {m}}-\int \Gamma (f, \Delta f)e^{-2w} \varphi \,{\mathrm {d}}{\mathfrak {m}}\Big \}}_{\Gamma _2(f; e^{-2w} \varphi )}+\Big (\int \Gamma \big (w, e^{-2w}\Gamma (f)\varphi \big )\,{\mathrm {d}}{\mathfrak {m}}\\&\qquad -\int \Gamma \big (w, e^{-2w}\Gamma (f)\big )\varphi \,{\mathrm {d}}{\mathfrak {m}}\Big ) - \int \Gamma \big ( \Gamma (f), w\big )e^{-2w} \varphi \,{\mathrm {d}}{\mathfrak {m}}+2\int \Delta f\Gamma (f, w) e^{-2w} \varphi \,{\mathrm {d}}{\mathfrak {m}}\\&\quad =\Gamma _2(f; e^{-2w} \varphi )+\int \Gamma \big (w, e^{-2w}\Gamma (f)\varphi \big )\,{\mathrm {d}}{\mathfrak {m}}-\Big (\int \Gamma \big (w, \Gamma (f)\big )\varphi e^{-2w}\,{\mathrm {d}}{\mathfrak {m}}-2\int \Gamma (w)\Gamma (f)e^{-2w}\varphi \,{\mathrm {d}}{\mathfrak {m}}\Big )\\&\qquad - \int \Gamma \big ( \Gamma (f), w\big )e^{-2w} \varphi \,{\mathrm {d}}{\mathfrak {m}}+2\int \Delta f\Gamma (f, w) e^{-2w} \varphi \,{\mathrm {d}}{\mathfrak {m}}\\&\quad = {\hbox {(I) + (II) + (III) + (IV)}}-\Big [\int \frac{(N-2)(N'-2)}{N'-N} \Gamma (w,f)^2 e^{-2w} \varphi \,{\mathrm {d}}{\mathfrak {m}}+\int \overline{ \varphi \Gamma ^w(f)} \,{\mathrm {d}}{\varvec{\Delta }} w \Big ] \end{aligned}$$

where

$$\begin{aligned} {\hbox {(I)}}= & {} \Gamma _2(f; e^{-2w} \varphi )- \int \Big (|{\mathrm {Hess}}_f|^2_\mathrm{HS} +\frac{1}{N-\dim _{\mathrm{loc}}}( \Delta f-{\mathrm{{tr}}}{\mathrm {Hess}}_f)^2\Big )\varphi e^{-2w}\,{\mathrm {d}}{\mathfrak {m}},\\ {\hbox {(II)}}= & {} \int \Big {(}|{\mathrm {Hess}}_f|^2_{\mathrm{HS}}+2\Gamma (f)\Gamma (w)+({\dim _{\mathrm{loc}}}-2) \Gamma (f,w)^2-2\Gamma \big (w,\Gamma (f)\big )\\&+2\Gamma (f,w){\mathrm{{tr}}}{\mathrm {Hess}}_f \Big {)}\varphi e^{-2w}\,{\mathrm {d}}{\mathfrak {m}},\\ {\hbox {(III)}}= & {} \frac{1}{N'-\dim _{\mathrm{loc}}}\int \Big {(} \Delta f-{\mathrm{{tr}}}{\mathrm {Hess}}_f+(2-\dim _{\mathrm{loc}}) \Gamma (f,w)\Big {)}^2\varphi e^{-2w}\,{\mathrm {d}}{\mathfrak {m}}, \end{aligned}$$

and

$$\begin{aligned} {\hbox {(IV)}}= & {} \int \bigg [ \Big (\frac{1}{N-\dim _{\mathrm{loc}}}-\frac{1}{N'-\dim _{\mathrm{loc}}}\Big ) (\Delta f-{\mathrm{{tr}}}{\mathrm {Hess}}_f)^2\\&+2\Big (1-\frac{2-\dim _{\mathrm{loc}}}{N'-\dim _{\mathrm{loc}} }\Big ) (\Delta f-{\mathrm{{tr}}}{\mathrm {Hess}}_f)\Gamma (f,w)\\&+\Big ( \frac{(N-2)(N'-2)}{N'-N} -( \dim _{\mathrm{loc}}-2)-\frac{( \dim _{\mathrm{loc}}-2)^2}{N'-\dim _{\mathrm{loc}}}\Big )\Gamma (f,w)^2 \bigg ]\varphi e^{-2w}\,{\mathrm {d}}{\mathfrak {m}}. \end{aligned}$$

By Proposition 4 we obtain

$$\begin{aligned} {\hbox {(I)}} \ge \int {k} \Gamma (f) \overline{e^{-2w}\varphi }\,{\mathrm {d}}{\mathfrak {m}}. \end{aligned}$$

By Lemma 4, we get

$$\begin{aligned} {\hbox {(II) + (III)}} \ge \frac{1}{N'}\int \big {(} \Delta f\big {)}^2\varphi e^{-2w}\,{\mathrm {d}}{\mathfrak {m}}. \end{aligned}$$

As for the last term (IV), it can be checked that the function in the bracket is positive definite, so (IV) $\ge 0$.

Combining the computations above, we complete the proof. $\square$

#### **Lemma 4**

*For any* $f \in \mathrm{TestF}_{\mathrm{{loc}}}({\mathcal {E}})$, *we have*

$$\begin{aligned} A_1+\frac{1}{N'-\dim _{\mathrm{loc}}}A_2^2\ge \frac{1}{N'}\big {(} \Delta f\big {)}^2~~{\mathfrak {m}}{\text {-a.e.}} \end{aligned}$$

*where*

$$\begin{aligned} A_1:= & {} |{\mathrm {Hess}}_f|^2_{\mathrm{HS}}+2\Gamma (f)\Gamma (w)+({\dim _{\mathrm{loc}}}-2) \Gamma (f,w)^2\\- & {} 2\Gamma (w,\Gamma (f))+2\Gamma (f,w){\mathrm{{tr}}}{\mathrm {Hess}}_f \end{aligned}$$

*and*

$$\begin{aligned} A_2:= \Delta f-{\mathrm{{tr}}}{\mathrm {Hess}}_f-({\dim _{\mathrm{loc}}}-2) \Gamma (f,w). \end{aligned}$$

#### *Proof*

By Proposition 3, there exists an orthonormal basis $\{e_i\}_i \subset L^2(TX)$. Then, we denote ${\mathrm {Hess}}_f(e_i, e_j)$ by $({\mathrm {Hess}}_f)_{ij}$ and denote ${\langle }\nabla g, e_i {\rangle }$ by $g_i$ for any $g\in {\mathbb {V}}^e$. We define a matrix $H:=(H_{ij})$ by $H_{ij}=({\mathrm {Hess}}_f)_{ij}-w_if_j-w_jf_i+\Gamma (f,w)\delta _{ij}$. Then, we have

$$\begin{aligned} \mathop {\sum }_{i,j}H^2_{ij}= & {} \mathop {\sum }_{i,j}\big {(}({\mathrm {Hess}}_f)_{ij}-w_if_j-w_jf_i+\Gamma (f,w)\delta _{ij} \big {)}^2\\= & {} |{\mathrm {Hess}}_f|^2_{\mathrm{HS}} +2\Gamma (f)\Gamma (w)+({\dim _{\mathrm{loc}}}-2) \Gamma (f,w)^2\\&~~~-2\Gamma (w,\Gamma (f))+2\Gamma (f,w){\mathrm{{tr}}}{\mathrm {Hess}}_f \ = \ A_1 \end{aligned}$$

where ${\mathrm{{tr}}}{\mathrm {Hess}}_f=\mathop {\sum }_{i} ({\mathrm {Hess}}_f)_{ii}$. It can also be seen that

$$\begin{aligned} {\mathrm{{tr}}}H =\mathop {\sum }_{i}H_{ii}={\mathrm{{tr}}}{\mathrm {Hess}}_f+({\dim _{\mathrm{loc}}}-2) \Gamma (f,w)=\Delta f-A_2. \end{aligned}$$

Finally, we obtain

$$\begin{aligned} A_1+\frac{1}{N'-\dim _{\mathrm{loc}}}A_2^2= & {} \Vert H\Vert ^2_{\mathrm{HS}}+\frac{1}{N'-\dim _{\mathrm{loc}}}\Big ( {\mathrm{{tr}}}H-\Delta f \Big )^2\\\ge & {} \frac{1}{\dim _{\mathrm{loc}}}\big ( {\mathrm{{tr}}}H \big )^2+\frac{1}{N'-\dim _{\mathrm{loc}}}\Big ( {\mathrm{{tr}}}H-\Delta f \Big )^2\ \ge \frac{1}{N'} \Big ( \Delta f \Big )^2 \end{aligned}$$

which is the thesis. $\square$

#### **Theorem 5**

(${\text {BE}}_{\mathrm{{loc}}}({k},N)$ condition under time change) *Let* ${\mathcal {E}}$ *be a Dirichlet form satisfying the* ${\text {BE}}_{\mathrm{{loc}}}({k},N)$ *condition for some* $N\in [2, \infty )$ and ${k} \in L_{\mathrm{{loc}}}^\infty (X, {\mathfrak {m}})$. *Assume that* $w\in {D}_{\mathrm{{loc}}}({\varvec{\Delta }}) \cap L_{\mathrm{{loc}}}^\infty$ with ${\varvec{\Delta }} w={\varvec{\Delta }}_\mathrm{{sing}} w+{ \Delta }_\mathrm{{ac}} w \, {\mathfrak {m}}$ *and* ${\varvec{\Delta }}_\mathrm{{sing}} w \le 0$. *Moreover, assume that for some* $N'\in (N, \infty ]$ *and* $k'\in L^\infty _{\mathrm{{loc}}}$ *it holds*

10 $$\begin{aligned} k'\le e^{-2w}\Big [{k}- \frac{(N-2)(N'-2)}{N'-N} \Gamma (w)- {\Delta }_\mathrm{{ac}} w \Big ] \end{aligned}$$

${\mathfrak {m}}$*-a.e. on* *X*. *Then, the time-changed Dirichlet form* ${\mathcal {E}}^w$ *on* $L^2(X,{\mathfrak {m}}^w)$ *satisfies the* ${\text {BE}}_{\mathrm{{loc}}}(k', N')$ *condition.*

*In particular, if there are some* $N'\in (N, \infty ]$ *and* $K'\in {\mathbb {R}}$ *such that*

11 $$\begin{aligned} K'\le e^{-2w}\Big [{k}- \frac{(N-2)(N'-2)}{N'-N} \Gamma (w)- {\Delta }_\mathrm{{ac}} w \Big ] \end{aligned}$$

${\mathfrak {m}}$-a.e. on *X*, *we have the following gradient estimate*

$$\begin{aligned} \Gamma ^w(P^w_t f) +\frac{1-e^{-2K't}}{N'K'} (\Delta ^w P_t^w f)^2\le e^{-2K't} P^w_t \big (\Gamma ^w(f)\big )~~~~~~{\mathfrak {m}}^w{\text {-a.e.}} \end{aligned}$$

*for all* $$f\in {D}({\mathcal {E}}^w)$$.

#### *Proof*

Given the estimate (9) from the previous theorem, we iteratively will extend the class of functions for which it holds true.

(i) Our first claim is that (9) holds for all $f\in {D}_{\mathrm{{loc}}}(\Delta )\cap L^\infty _{\mathrm{{loc}}}$ with $\Delta f\in {\mathbb {V}}_{\mathrm{{loc}}}$ and all compactly supported, nonnegative $\varphi \in {\mathbb {V}}^\infty$ with $\Gamma (\varphi )\in L^\infty$. Indeed, given such *f* and $\varphi$, choose $f'\in {D}(\Delta )\cap L^\infty$ with $\Delta f\in {\mathbb {V}}$ such that $f=f'$ on a neighborhood of $\{\varphi \not =0\}$. Choose $f_n\in \mathrm{TestF}({\mathcal {E}})$ with $f_n\rightarrow f'$ in ${D}(\Delta )$ and $\Delta f_n\rightarrow \Delta f'$ in ${\mathbb {V}}$. (For instance, put $f_n=P_{1/n}f'$.) Applying (9) with $f_n$ in the place of *f* and passing to the limit $n\rightarrow \infty$ yields the claim. Indeed,

$$\begin{aligned} \Gamma ^w(\Gamma ^w(f_n),\varphi )=e^{-4w}\Big [\Gamma (\Gamma (f_n),\varphi )-2\Gamma (f_n)\cdot \Gamma (w,\varphi )\Big ] \end{aligned}$$

which according to Lemma 1 for $n\rightarrow \infty$ converges to

$$\begin{aligned} e^{-4w}\Big [\Gamma (\Gamma (f),\varphi )-2\Gamma (f)\cdot \Gamma (w,\varphi )\Big ]=\Gamma ^w(\Gamma ^w(f),\varphi ) \end{aligned}$$

since $$f_n\rightarrow f$$ in $${D}(\Delta )$$.

(ii) Our next claim is that (9) holds for all $f\in {D}_{\mathrm{{loc}}}(\Delta ^w)\cap L^\infty _{\mathrm{{loc}}}({\mathfrak {m}}^w)$ with $\Delta ^w f\in ({\mathbb {V}}^w)_{\mathrm{{loc}}}^\infty$ and all compactly supported, nonnegative $\varphi \in ({\mathbb {V}}^w)^\infty$ with $\Gamma ^w(\varphi )\in L^\infty ({\mathfrak {m}}^w)$. Indeed, the conditions on *f* and on $\varphi$ will not depend on *w* as long as $w\in {\mathbb {V}}_{\mathrm{{loc}}}^\infty$ which is the case by assumption. This is obvious in the case of the conditions on $\varphi$. For the conditions on *f*, note that $\Delta ^w=e^{-2w}\Delta$ and $\Gamma ^w(\Delta ^w)\le 2e^{-4w}\big [\Gamma (\Delta f)+4(\Delta f)^2\, \Gamma (w)\big ]$.

(iii) Taking into account the assumptions on ${\varvec{\Delta }} w$ and on $k'$, according to Lemma 2 together with Remark 4 the assertion of the second claim already proves ${\text {BE}}_{\mathrm{{loc}}}(k',N')$.

(iv) The gradient estimate is a standard consequence of $\mathrm{BE}(K',N')$, see [10]. $\square$

#### *Remark 5*

Let $e^{2w}=\rho$ and $N'=\infty$ in Theorem 5. Then, condition (11) becomes

$$\begin{aligned} K'\le K\rho ^{-1}+\frac{1}{2} \Delta \rho ^{-1}-N\Gamma (\rho ^{-\frac{1}{2}})=\rho ^{-\frac{N}{2}}\Big ( K-\frac{1}{N-2} \Delta \Big )\rho ^{\frac{N}{2}-1}. \end{aligned}$$

Furthermore, when $N=2$, the condition is $K'\rho \le K-\frac{1}{2} \Delta \ln \rho$.

## Time change and the Lott–Sturm–Villani condition

In this section, we will study synthetic lower Ricci bounds under time change in the setting of metric measure spaces. More precisely, we will derive the transformation formula for the curvature-dimension condition of Lott–Sturm–Villani under time change.

### Metric measure spaces and time change

#### **Assumption 2**

In this section we will assume that the metric measure space $(X,{\mathrm {d}},{\mathfrak {m}})$ fulfils the following conditions:

(i) $(X,{\mathrm {d}})$ is a complete and separable geodesic space;
(ii) ${\mathfrak {m}}$ is a ${\mathrm {d}}$-Borel measure and $\mathop {\mathrm{{supp}}}\nolimits {{\mathfrak {m}}}=X$;
(iii) $(X,{\mathrm {d}},{\mathfrak {m}})$ satisfies the Riemannian curvature-dimension condition ${\mathrm{{RCD}}}(K, N)$ for some $K\in {\mathbb {R}}$ and $N\in [1, \infty )$.

Given such a metric measure space $(X,{\mathrm {d}},{\mathfrak {m}})$, the energy is defined on $L^2(X,{\mathfrak {m}})$ by

$$\begin{aligned} {\mathcal {E}}(f):= & {} \inf \!\Big \lbrace \liminf _{n \rightarrow \infty } \int _X {\mathrm {lip}}({f_n})^2{\mathrm {d}}{\mathfrak {m}}: f_n \in \mathop {\mathrm{{Lip}}}\nolimits _{b}(X),\ \! f_n \rightarrow f {\text { in }} L^2(X,{\mathfrak {m}})\Big \rbrace \\= & {} \int _X\big | \mathrm {D}f|^2\,{\mathrm {d}}{\mathfrak {m}}\end{aligned}$$

where ${\mathrm {lip}}({f})(x) := \limsup _{y\rightarrow x} \vert f(x) - f(y)\vert /\! {\mathrm {d}}(x,y)$ denotes the *local Lipschitz slope* and $|\mathrm {D}f|(x)$ denotes the minimal weak upper gradient at $x\in X$. We refer to [1] for details. As a part of the definition of RCD condition, ${\mathcal {E}}(\cdot )$ is a quadratic form. By polarization, this defines a quasi-regular, strongly local, conservative Dirichlet form admitting a carré du champ $\Gamma (f):=|\mathrm {D}f|^2$. We use the notations $W ^{1,2}(X,{\mathrm {d}},{\mathfrak {m}})={\mathbb {V}}={D}({\mathcal {E}})$ and $S^2(X,{\mathrm {d}},{\mathfrak {m}})={\mathbb {V}}^e$.

#### **Definition 5**

Given $w\in L^2_{\mathrm{{loc}}}(X,{\mathfrak {m}})$, the time-changed metric measure space is defined as $(X, {\mathrm {d}}^w, {\mathfrak {m}}^w)$ where ${\mathfrak {m}}^w:=e^{2w}{\mathfrak {m}}$ and ${\mathrm {d}}^w$ is given by

12 $$\begin{aligned} {\mathrm {d}}^w(x, y):=\sup \big \{\phi (x)-\phi (y): \phi \in {\mathbb {V}}_{\mathrm{{loc}}}\cap C(X), |\mathrm {D}\phi | \le e^w \ {\mathfrak {m}}{\text {-a.e. in}}~ X \big \} \end{aligned}$$

for any $$x, y \in X$$.

#### *Remark 6*

There are various alternative definitions for the distance function under time change. The first of them is

13 $$\begin{aligned} {\mathrm {d}}_w(x, y):=\sup \big \{\phi (x)-\phi (y): \phi \in \mathop {\mathrm{{Lip}}}\nolimits _{\mathrm{{loc}}}(X), {\mathrm {lip}}({\phi })\le e^w \ {\mathfrak {m}}{\text {-a.e. in}}~ X \big \}. \end{aligned}$$

Since $\mathop {\mathrm{{Lip}}}\nolimits _{\mathrm{{loc}}}(X)\subset {\mathbb {V}}_{\mathrm{{loc}}}\cap C(X)$ and $|\mathrm {D}\phi |\le {\mathrm {lip}}({\phi })$ for $\phi \in \mathop {\mathrm{{Lip}}}\nolimits _{\mathrm{{loc}}}(X)$, obviously ${\mathrm {d}}_w\le {\mathrm {d}}^w$. It is easy to see that in both of these definitions, the class of functions under consideration can equivalently be restricted to those with compact supports. In other words, ${\mathrm {d}}^w(x, y)=\sup \big \{\phi (x)-\phi (y): \phi \in {\mathbb {V}}\cap C_c(X), |\mathrm {D}\phi | \le e^w\ {\mathfrak {m}}{\text {-a.e. in}}~ X \big \}$ and ${\mathrm {d}}_w(x, y)=\sup \big \{\phi (x)-\phi (y): \phi \in \mathop {\mathrm{{Lip}}}\nolimits _c(X), {\mathrm {lip}}({\phi })\le e^w\ {\mathfrak {m}}{\text {-a.e. in}}~ X \big \}$.

Moreover, we consider the metric $e^w\odot {\mathrm {d}}$ defined in a dual way by

14 $$\begin{aligned} \big (e^w\odot {\mathrm {d}}\big )(x,y):=\inf \Big \{\int _0^1 e^w (\gamma _s)\, |{\dot{\gamma }}_s|\,{\mathrm {d}}s: \gamma \in \mathrm{AC}([0,1], X), \gamma _0=x,\gamma _1=y\Big \}. \end{aligned}$$

Of particular interest is the metric $e^{{\bar{w}}}\odot {\mathrm {d}}$ with *w* replaced by its upper semicontinuous envelope ${\bar{w}}$ defined by

$$\begin{aligned} {\bar{w}}(x):=\limsup _{y\rightarrow x}w(y). \end{aligned}$$

#### **Lemma 5**

*Assume that* *w* *is continuous a.e. on* *X*. *Then, each of the metrics* ${\mathrm {d}}^w, {\mathrm {d}}_w$, $e^w\odot {\mathrm {d}}$ *and* $e^{{\bar{w}}}\odot {\mathrm {d}}$ *induces the same minimal weak upper gradient* $|\mathrm {D}^wf|=e^{-w}|\mathrm {D}f|$ ${\mathfrak {m}}$*-a.e. on* *X* *for each* $f\in L^2_{\mathrm{{loc}}}(X)$*. In particular,*

$$\begin{aligned} \Gamma ^w=e^{-2w}\, \Gamma \quad {\text {on }}{\mathbb {V}}_{\mathrm{{loc}}}={\mathbb {V}}_{\mathrm{{loc}}}^w \end{aligned}$$

*where here and henceforth* $\Gamma ^w$ *denotes the carré du champ operator induced by the metric measure space* $(X,{\mathrm {d}}^w,{\mathfrak {m}}^w)$.

#### *Proof*

Assume that *w* is continuous at $x\in X$. Then, for each $\varepsilon >0$ there exists $\delta >0$ such that $|w(x)-w(y)|<\varepsilon$ for $y\in B_\delta (x)$. Hence, by using appropriate truncation arguments it is easy to see that for each ${\mathrm {d}}^*\in \big \{ {\mathrm {d}}^w, {\mathrm {d}}_w, e^w\odot {\mathrm {d}}, e^{{\bar{w}}}\odot {\mathrm {d}}\big \}$ and all $y\in B_\delta (x)$

$$\begin{aligned} e^{w(x)-\varepsilon }\cdot {\mathrm {d}}(x,y)\quad \le \quad {\mathrm {d}}^*(x,y) \quad \le \quad e^{w(x)+\varepsilon }\cdot {\mathrm {d}}(x,y). \end{aligned}$$

Hence, ${\mathrm {lip}}^*(f)(x)=e^{-w(x)}\,{\mathrm {lip}}(x)$ for the respective local Lipschitz constants associated with ${\mathrm {d}}^*$.

To obtain the respective minimal weak upper gradient for $f\in L^2(X,{\mathfrak {m}})$ associated with ${\mathrm {d}}^*$, one has to consider the relaxations of ${\mathrm {lip}}^*(f)$ w.r.t. the measure ${\mathfrak {m}}^w=e^{2w}{\mathfrak {m}}$. This, however, amounts to study the relaxations of the original ${\mathrm {lip}}(f)$ w.r.t. the measure ${\mathfrak {m}}$. Thus, the claimed identify $\Gamma ^w(f)=e^{-2w}\, \Gamma (f)$ ${\mathfrak {m}}$-a.e. on *X* follows. $\square$

In the following lemma, we show the coincidence of ${\mathrm {d}}^w$ and $e^{{\bar{w}}}\odot {\mathrm {d}}$, see [17] for related results.

#### **Lemma 6**

*Assume that* *w* *is continuous a.e. on* *X*. *Then,*

$$\begin{aligned} {\mathrm {d}}^w=e^{{\bar{w}}}\odot {\mathrm {d}}. \end{aligned}$$

*In particular,* ${\mathrm {d}}^w$ *is a geodesic metric.*

#### *Proof*

(i) Let us first prove that ${\mathrm {d}}^w$ is a geodesic metric. Since *X* is locally compact w.r.t. the metric ${\mathrm {d}}$ and since the metrics ${\mathrm {d}}^w$ and ${\mathrm {d}}$ are locally equivalent, the space *X* is also locally compact w.r.t. the metric ${\mathrm {d}}^w$. Therefore, it suffices to prove that ${\mathrm {d}}^w$ is a length metric. Assume this is not the case. Then, there exist points $x\not = y$ with ${\mathrm {d}}^w(x,y)<2r$ and $B^w_r(x)\cap B^w_r(y)=\emptyset$. Put

$$\begin{aligned} f={\mathrm {d}}^w(.,X{\setminus } B_r(x))-{\mathrm {d}}^w(.,X{\setminus } B_r(y)). \end{aligned}$$

It is easy to verify that $\Gamma ^w(f)\le 1$ and obviously *f* is continuous. Hence, by the very definition of ${\mathrm {d}}^w$

$$\begin{aligned} {\mathrm {d}}^w(x,y)\ge f(x)-f(y)=2r \end{aligned}$$

which is in contradiction to our initial assumption.

(ii) Now let us consider the particular case where *w* is continuous on all of *X*. Then, ${\mathrm {d}}^{w}=e^{w}\odot {\mathrm {d}}$. Indeed, both metrics are geodesic metrics on *X* and coincide up to multiplicative pre-factors $e^{\pm \varepsilon }$ on suitable neighborhoods $B_\delta (x)$ of each point $z\in X$.

(iii) To deal with the general case, let us choose a decreasing sequence of continuous functions $w_n$ with $w_n\downarrow {\bar{w}}$ as $n\rightarrow \infty$. Then, ${\mathrm {d}}^{w_n}=e^{w_n}\odot {\mathrm {d}}$ for each *n* by the preceding case ii) and thus by monotonicity for all *x*, *y*

$$\begin{aligned} {\mathrm {d}}^w(x,y)\le \inf _n {\mathrm {d}}^{w_n}(x,y)=\inf _n(e^{w_n}\odot {\mathrm {d}})(x,y)=(e^{{\bar{w}}}\odot {\mathrm {d}})(x,y). \end{aligned}$$

iv) To prove the reverse estimate, for given $x\in X$ observe that $f=(e^{{\bar{w}}}\odot {\mathrm {d}})(x,.)$ is continuous and obviously ${\mathrm {lip}}^w(f)(y)\le 1$ in each point *y* of continuity of *w*. Thus, in particular, $\Gamma ^w(f)\le 1$ ${\mathfrak {m}}^w$-a.e. on *X*. This indeed implies that ${\mathrm {d}}^w(x,z)\ge |f(x)-f(z)|=(e^{{\bar{w}}}\odot {\mathrm {d}})(x,z)$ for each $z\in X$. $\square$

#### **Lemma 7**

*Assume that* *w* *is continuous a.e. on* *X*. *Then, the metric measure space* $(X,{\mathrm {d}}^w,{\mathfrak {m}}^w)$ *has the Sobolev-to-Lipschitz property.*

#### *Proof*

Assume that $f\in {\mathbb {V}}_{\mathrm{{loc}}}$ is given with $\Gamma ^w(f)\le 1$ ${\mathfrak {m}}^w$-a.e. on *X*. By truncation one can achieve on each bounded set *B* that $f=f_B$ a.e. on *B* for some $f_B$ with bounded support and with $\Gamma ^w(f_B)\le 1$ ${\mathfrak {m}}^w$-a.e. on *X*. Since $w\in L^\infty _{\mathrm{{loc}}}$, moreover, $\Gamma (f_B)\le C$ ${\mathfrak {m}}$-a.e. on *X*. By the Sobolev-to-Lipschitz property of the original metric measure space $(X,{\mathrm {d}},{\mathfrak {m}})$ it follows that $f={\bar{f}}_B$ a.e. on *B* for some ${\bar{f}}_B$ with $\mathop {\mathrm{{Lip}}}\nolimits ({\bar{f}}_B)\le C$. In particular, $\bar{f}_B$ is continuous and $\Gamma ^w({\bar{f}}_B)\le 1$ ${\mathfrak {m}}^w$-a.e. on *X*. Hence,

$$\begin{aligned} {\mathrm {d}}^w(x,y)\ge |{\bar{f}}_B(x)-{\bar{f}}_B(y)| \end{aligned}$$

for all $x,y\in X$ by the very definition of the metric ${\mathrm {d}}^w$. In other words, ${\bar{f}}_B\in \mathop {\mathrm{{Lip}}}\nolimits ^w(X)$ with $\mathop {\mathrm{{Lip}}}\nolimits ^w({\bar{f}}_B)\le 1$. Considering these constructions for an open covering of *X* by such sets *B*, it follows that there exists ${\bar{f}}\in \mathop {\mathrm{{Lip}}}\nolimits ^w(X)$ with $f={\bar{f}}$ ${\mathfrak {m}}$-a.e. on *X* and $\mathop {\mathrm{{Lip}}}\nolimits ^w({\bar{f}})\le 1$. $\square$

Finally, we can prove the transformation formula for the ${\mathrm{{RCD}}}(K, N)$ condition under time change.

#### **Theorem 6**

*Let* $(X,{\mathrm {d}},{\mathfrak {m}})$ *be an* ${\mathrm{{RCD}}}(K, N)$ *space for some* $K\in {\mathbb {R}}$ *and* $N\in [1, \infty )$, *and let* $w\in {D}_{\mathrm{{loc}}}({\varvec{\Delta }})\cap L^\infty _{\mathrm{{loc}}}(X)$ *be continuous* ${\mathfrak {m}}$*-a.e. with* ${\varvec{\Delta }} w={\varvec{\Delta }}_\mathrm{{sing}} w+{ \Delta }_\mathrm{{ac}} w \, {\mathfrak {m}}$ *and* ${\varvec{\Delta }}_\mathrm{{sing}} w \le 0$. *Then, the time-changed metric measure space* $(X, {\mathrm {d}}^{w}, {\mathfrak {m}}^w)$ *satisfies the* $\mathrm{RCD}(K', N')$ *condition for any* $N'\in (N, \infty )$ *and* $K'\in {\mathbb {R}}$ *such that* ${\mathfrak {m}}$*-a.e. on* *X*

15 $$\begin{aligned} K'\le e^{-2w}\Big [K- \frac{(N-2)(N'-2)}{N'-N} |\mathrm {D}w|^2- { \Delta }_\mathrm{{ac}} w \Big ]. \end{aligned}$$

A particular consequence of the theorem is that the time-changed metric measure space $(X,{\mathrm {d}}^w,{\mathfrak {m}}^w)$ satisfies the squared exponential volume growth condition:

$$\exists C\in {\mathbb {R}}, z\in X$$:

16 $$\begin{aligned} {\mathfrak {m}}^w(B^w_r(z))\le C \, e^{Cr^2}\qquad (\forall r>0). \end{aligned}$$

#### *Proof*

From the work of [3, 10], we know that the curvature-dimension condition RCD(*K*, *N*) implies the Bakry–Émery condition BE(*K*, *N*) for the Dirichlet form ${\mathcal {E}}$ on $L^2(X,{\mathfrak {m}})$ induced by the measure space $(X,{\mathrm {d}},{\mathfrak {m}})$. According to Theorem 5, this implies the Bakry–Émery condition BE$(K',N')$ for the Dirichlet form ${\mathcal {E}}^w$ on $L^2(X,{\mathfrak {m}}^w)$. Due to Lemma 5, the latter indeed is the Dirichlet form induced by the metric measure space $(X,{\mathrm {d}}^w,{\mathfrak {m}}^w)$. Finally, again by [3, 10], BE$(K',N')$ for the Dirichlet form induced by $(X,{\mathrm {d}}^w,{\mathfrak {m}}^w)$ will imply ${\hbox {RCD}}^*(K',N')$ provided the volume-growth condition (16) is satisfied and the Sobolev-to-Lipschitz property holds. The latter was proven in Lemma 7. To deal with the former, we proceed in two steps.

i) Let us first consider the case $w\in L^\infty (X)$. Then, the volume-growth condition (16) for $(X,{\mathrm {d}}^w,{\mathfrak {m}}^w)$ obviously fellows from that for $(X,{\mathrm {d}},{\mathfrak {m}})$ which in turn follows from the ${\mathrm{{RCD}}}(K, N)$ assumption.

ii) Now let general $w\in L_{\mathrm{{loc}}}^\infty (X)$ be given as well as $K'$ and $N'$ such that (15) is satisfied. Given $z\in X$, define $w_l=w\cdot \chi _\ell$ with suitable cut-off functions $(\chi _\ell )_{\ell \in {\mathbb {N}}}$ (cf. [4], Lemma 6.7) such that for all $\ell \in {\mathbb {N}}$

- $$w_\ell =w$$ on $$B_\ell (z)$$
- $w_\ell$ is bounded on *X*
- $$e^{-2w_\ell }\Big [K- \frac{(N-2)(N'-2)}{N'-N} |\mathrm {D}w_\ell |^2- { \Delta }_\mathrm{{ac}} w_\ell \Big ]\ge K'-1$$.

Then, according to part i) of this proof, the metric measure space $(X,{\mathrm {d}}^{w_\ell },{\mathfrak {m}}^{w_\ell })$ satisfies $\mathrm{RCD}(K'-1,N')$. This in particular implies that there exists a constant *C* (which indeed can be chosen independent of $\ell$) such that

$$\begin{aligned} {\mathfrak {m}}^{w_\ell }\big (B_r^{w_\ell }(z)\big )\le C\, e^{C\, r^2} \end{aligned}$$

for all $r>0$. Since ${\mathfrak {m}}^{w_\ell }\big (B_r^{w_\ell }(z)\big )={\mathfrak {m}}^{w}\big (B_r^{w}(z)\big )$ for all $r\le \ell$, this finally proves the requested volume growth condition. $\square$

It might be of certain interest to analyze the validity of the volume growth condition (16) under time change without referring to curvature bounds. We will still assume that *w* is ${\mathfrak {m}}$-a.e. continuous.

#### **Lemma 8**

*Suppose there exist non-negative* $p,q\in L^\infty _{\mathrm{{loc}}}({\mathbb {R}}_+)$ *with*

$$\begin{aligned} -q({\mathrm {d}}(\cdot , x_0)) \le w(.)\le p({\mathrm {d}}(\cdot , x_0))\qquad {\mathfrak {m}}{\text {-a.e. on }}X. \end{aligned}$$

*Then,* $(X, {\mathrm {d}}^w, {\mathfrak {m}}^w)$ *satisfies the squared exponential volume growth condition:* $\exists C\in {\mathbb {R}}, x_0\in X$:

17 $$\begin{aligned} {\mathfrak {m}}^w(B^w_r(x_0))\le C \, e^{Cr^2}\qquad (\forall r>0) \end{aligned}$$

*provided the function* $f(r):=\int _0^r e^{-q(s)}{\text {d}}s$ *satisfies*

(i) $$\liminf _{r\rightarrow \infty } \frac{1}{r} f(r)>0$$ *and*
(ii) $$\limsup _{r\rightarrow \infty } \frac{1}{r^2}p\big (f^{-1}(r)\big )<\infty$$.

*In particular, if* *q* *is bounded and* $\mathop {\overline{\lim }}_{{r} \rightarrow {\infty }} \frac{p(r)}{r^2}<\infty$, *then* $(X, {\mathrm {d}}^w, {\mathfrak {m}}^w)$ *satisfies the squared exponential volume growth condition.*

#### *Proof*

From Lemma 6, we know

$$\begin{aligned} {\mathrm {d}}^w(x_0, y) \ge \int _0^ {{\mathrm {d}}(x_0, y)} \exp \big ( -q(r) \big )\,{\mathrm {d}}r=f({\mathrm {d}}(x_0, y)) \end{aligned}$$

for any $y\in X$. Since $f^{-1}$ is strictly increasing, this implies

$$\begin{aligned} B_{{R}}^{{w}}(x_0) \subset B_{f^{-1}{(R)}}(x_0),~~~~\forall ~R>0. \end{aligned}$$

Hence,

$$\begin{aligned} {\mathfrak {m}}^w \big (B_{R}^{w}(x_0)\big ) \le \exp \big (2p( f^{-1}(R))\big ){\mathfrak {m}}\big (B_{{f^{-1}(R)}}(x_0)\big ). \end{aligned}$$

Recall that the ${\mathrm{{RCD}}}(K, \infty )$ condition implies the squared exponential volume growth condition, so there exist $M, c >0$ such that

$$\begin{aligned} {\mathfrak {m}}^w \big (B_{R}^{{w}}(x_0)\big ) \le M\exp \Big (2p( f^{-1}(R))+c \big ( f^{-1}(R) \big )^2\Big ). \end{aligned}$$

Note that (i) implies $\limsup _{r\rightarrow \infty } \frac{1}{r} f^{-1}(r)<\infty$. Hence, together with (ii), this implies the squared exponential volume growth condition for $(X, {\mathrm {d}}^w, {\mathfrak {m}}^w)$. $\square$

### Convexity transform

Firstly we introduce the notion of local $\ell$-convexity (i.e., semi-convexity) in non-smooth setting. Such notion is derived from [18] by the second author and Lierl (see Definition 2.6 and Definition 2.9 therein).

#### **Definition 6**

($\ell$*-convex functions, Definition* 2.6 [18]) Given $\ell \in {\mathbb {R}}$, we say that a function *V* is $\ell$-convex on a closed subset $Z \subset X$ if there exists a convex open covering $\cup _i X_i \supset Z$ such that each $V{|_{X_i}}: \overline{X_i} \rightarrow (-\infty , +\infty ]$ is $\ell$-geodesically convex, in the sense that for each $x_0, x_1 \in \overline{X_i}$, there exists a geodesic $\gamma :[0, 1] \rightarrow X$ from $x_0$ to $x_1$ such that

$$\begin{aligned} V(\gamma (t)) \le (1-t)V(\gamma (0))+tV(\gamma (1))-\frac{\ell }{2}t(1-t)|\dot{\gamma }_t|^2,~~\forall t\in [0, 1]. \end{aligned}$$

#### **Definition 7**

(Locally $\ell$-convex sets, Definition 2.9 [18]) Let $\Omega \subset X$ be an open subset and let $V:= {\mathrm {d}}(., \Omega )-{\mathrm {d}}(., {X{\setminus }\Omega })$ denote the signed distance from the boundary, in the sequel also briefly denoted by $\pm {\mathrm {d}}(., \partial \Omega )$.

Given $ell<0$, we say that $\Omega$ is locally $\ell$-convex if for each $\delta > 0$ there exists $r > 0$, such that *V* is $(\ell -\delta )$-convex and $-V$ is 0-convex on $\Omega ^r_{-r}$ with $|\mathrm {D}V|\ge 1-\delta$ where $\Omega ^r_{-r}:= \{-r< V < r\}$.

#### *Remark 7*

Assume that *X* is a smooth Riemannian manifold, and $\Omega$ is a bounded open subset of *X* with smooth boundary. It is proved in Proposition 2.10 [18] that the real-valued second fundamental form on $\partial \Omega$ is negative and bounded from below by $\ell$ if and only if $\Omega$ is locally $\ell$-convex.

#### *Remark 8*

If $\Omega =\{ V<0\}$ for some 0-geodesically convex function *V* (i.e., a geodesically convex function), then $\Omega$ is geodesically convex in $(X, {\mathrm {d}})$. In order to study convex transform, we just need to analyze the ‘pure concave’ part of $\partial \Omega$. This is the reason why we require that $-V$ is 0-convex in Definition 7. Moreover, this condition is necessary in the proof of Lemma 11 below.

Then, we can convexify locally $\ell$-convex sets using time change and the following convexification technique developed in [18] (see Theorem 2.17 therein).

#### **Lemma 9**

(Convexification Theorem) *Let* $\Omega$ *be a locally* $\ell$*-convex subset in* *X* *for some* $\ell <0$. *Then,* $\Omega$ *is locally geodesically convex in* $(X, {\mathrm {d}}^{-\ell ' V})$ *for any* $\ell '<\ell$.

Next we recall some important results concerning $L^1$-optimal transport and measure decomposition. This theory has proven to be a powerful tool in studying the fine structure of metric measure spaces. We refer the readers to the lecture note [6] for an overview of this topic and the bibliography.

#### **Lemma 10**

(Localization for ${\mathrm{{RCD}}}(K, N)$ spaces, Theorem 3.8 and Theorem 5.1 [8]) *Let* $(X,{\mathrm {d}},{\mathfrak {m}})$ *be an essentially non-branching metric measure space with* $\mathop {\mathrm{{supp}}}\nolimits {\mathfrak {m}}=X$, *and satisfying* ${\mathrm{{RCD}}}(K, N)$ *condition for some* $K\in {\mathbb {R}}$ *and* $N \in (1, \infty )$. *Then, for any 1-Lipschitz function* *u* on *X* *and the transport set* ${\mathsf {T}}_u$ *associated with* *u* *(up to*${\mathfrak {m}}$*-measure zero set,* ${\mathsf {T}}_u$ *coincides with* $\{|\nabla u|=1\}$)*, there is a disjoint family of unparameterized geodesics* $\{X_q\}_{q \in {\mathfrak {Q}}}$ *such that*

18 $$\begin{aligned} {\mathfrak {m}}({\mathsf {T}}_u {\setminus } \cup X_q)=0, \end{aligned}$$

*and there is a probability measure* ${\mathfrak {q}}$ *on* ${\mathfrak {Q}}$ *such that*

19 $$\begin{aligned} {\mathfrak {m}}{|_{{\mathsf {T}}_u}}=\int _{{\mathfrak {Q}}} {\mathfrak {m}}_q\, {\mathrm {d}}{\mathfrak {q}}(q),~~{\mathfrak {q}}({\mathfrak {Q}})=1~~{\text {and}}~~{\mathfrak {m}}_q(X_q)=1~~{\mathfrak {q}}{\text {-a.e.}}~ q \in {\mathfrak {Q}}. \end{aligned}$$

*Furthermore, for* ${\mathfrak {q}}$*-a.e.* $q\in {\mathfrak {Q}}$, ${\mathfrak {m}}_q$ *is a Radon measure with* ${\mathfrak {m}}_q \ll {\mathcal {H}}^1 {|_{X_q}}$ *and* $(X_q, {\mathrm {d}}, {\mathfrak {m}}_q)$ *satisfies* ${\mathrm{{RCD}}}(K, N)$. *In particular,* ${\mathfrak {m}}_q=h_q {\mathcal {H}}^1 {|_{X_q}}$ *for some* ${\mathrm{{CD}}}(K, N)$ *probability density* $h_q$.

#### **Lemma 11**

*Let* *Y* *be a locally* $\ell$*-convex domain in* *X* *for some* $ell<0$*, and* ${\mathfrak {m}}(\partial Y)=0$. *Then, the transport set* $\mathsf T_V$ *associated with the signed distance function* *V* *has full measure in* *X*. *There is a disjoint family of unparameterized geodesics* $\{X_q\}_{q \in {\mathfrak {Q}}}$ *satisfying* (18) *and* (19) *in Lemma* 10, *and a constant* $r_0>0$ *such that*

$$\begin{aligned} V(a_q) \ge 0,~~~V(b_q)\le -r_0~~~ {\mathfrak {q}}{\text {-a.e.}}~q\in {\mathfrak {Q}} \end{aligned}$$

*where* $a_q=a_q(X_q), b_q=b_q(X_q)$ *are the end points of* $X_q$.

#### *Proof*

Firstly, recall that ${\mathrm{{RCD}}}(K, N)$ condition yields local compactness, so for any $x\in X{\setminus } \partial Y$, there is $z\in \partial Y$ such that ${\mathrm {d}}(x, z)={\mathrm {d}}(x, \partial Y)$ and thus $x\in \mathsf T_V$. So ${\mathfrak {m}}(X {\setminus } {\mathsf {T}}_V)=0$.

Secondly, by Definition 7, *V* is semi-convex on $Y^{0}_{-r_0}$ for some $r_0>0$. By the main theorem of [25], for each $x_0 \in Y^{0}_{-r_0}$ there exists a unique gradient flow for *V* (and $-V$) starting in $x_0$. In particular, there is a maximal transport (geodesic) line $\gamma \subset {\mathsf {T}}_V$ satisfying $V(\gamma _1)-V(\gamma _0)={\mathrm {d}}(\gamma _0, \gamma _1)$, $V(\gamma _1) \ge V(x_0)$ and $V(\gamma _0) \le -r_0$.

By Theorem 10 there is a disjoint family of unparameterized geodesics $\{X_q\}_{q \in {\mathfrak {Q}}}$ such that ${\mathfrak {m}}({\mathsf {T}}_V {\setminus } \cup X_q)=0$. In addition, $X_q \cap \{V \le -r_0\} \ne \emptyset$ and $X_q \cap \{V \ge 0\} \ne \emptyset$ for any $q \in {\mathfrak {Q}}$. Therefore ${\mathfrak {m}}(X {\setminus } \cup X_q)=0$, $V(a_q) \ge 0$ and $V(b_q)\le -r_0$. $\square$

#### **Proposition 5**

(Convexification) *Let* $\Omega$ *be a locally* $\ell$-convex *domain in* $(X, {\mathrm {d}})$ *for some* $\ell < 0$, *and* ${\mathfrak {m}}(\partial \Omega )=0$. *Then, for any* $\ell '<\ell$, *there exist* $r_0>0$ *and a Lipschitz function* *w* *such that* $\Omega$ *is locally geodesically convex in* $(X, {\mathrm {d}}^{w})$ *and* $w \in {D}({\varvec{\Delta }}, X {\setminus } \partial \Omega )$ *with*

$$\begin{aligned} {\varvec{\Delta }} w{|_{X {\setminus } \partial \Omega }} \le -\ell ' \Big (\cot _{K, N}( r_0/4) +\frac{2}{ r_0}\Big ){\mathfrak {m}}{|_{X {\setminus } \partial \Omega }}. \end{aligned}$$

*where the function* $\cot _{K, N}: [0, +\infty ) \rightarrow [0, +\infty )$ *is defined by*

$$\begin{aligned} \cot _{K, N}(x):=\left\{ \begin{array}{lll} \sqrt{K(N-1)} \cot \left( \sqrt{\frac{K}{N-1}} x\right) , &{}{\text {if}}~~ K>0,\\ ({N-1})/x, &{}{\text {if}}~~K=0,\\ \sqrt{-K(N-1)} \coth \left( \sqrt{\frac{-K}{N-1}} x\right) , &{}{\text {if}} ~~K<0. \end{array} \right. \end{aligned}$$

#### *Proof*

Let $V:=\pm {\mathrm {d}}(\cdot , \partial Y)$ be the signed distance from the boundary and $r_0>0$ be the constant in Lemma 11. Given $\ell '<\ell \le 0$, we can find a smooth cut-off function $\phi : {\mathbb {R}}\rightarrow [0, r_0]$ satisfying

$$\begin{aligned} \phi (t) :=\left\{ \begin{array}{lll} t, &{}{\text {if}}~~ t\in [\frac{1}{4} \ell ' r_0, -\frac{1}{4} \ell 'r_0]\\ -\frac{1}{2} \ell 'r_0, &{}{\text {if}}~~ t\in [ -\frac{3}{4} \ell 'r_0, +\infty )\\ \frac{1}{2} \ell 'r_0, &{}{\text {if}}~~ t\in (-\infty , \frac{3}{4} \ell ' r_0] \end{array} \right. \end{aligned}$$

with $0\le \phi '\le 1$, $| \phi ''|\le -\frac{2}{\ell ' r_0}$ on ${\mathbb {R}}$. Then, we define $w:=\phi (-\ell 'V)$. By Convexification Theorem (cf. Theorem 2.17 [18]) we know $\Omega$ is locally geodesically convex in $(X, {\mathrm {d}}^{w})$.

By chain rule (cf. Proposition 4.11 [13]) and Corollary 4.16 [9] we have $w \in {D}({\varvec{\Delta }}, X {\setminus } \partial \Omega )$, and

20 $$\begin{aligned} {\varvec{\Delta }} w{|_{X {\setminus } \partial \Omega }}= & {} -\ell '\phi '(-\ell 'V) {\varvec{\Delta }} V{|_{X {\setminus } \partial \Omega }}+(\ell ')^2\phi ''(-\ell 'V) |\mathrm {D}V|^2\,{\mathfrak {m}}{|_{X {\setminus } \partial \Omega }}. \end{aligned}$$

In addition, by Corollary 4.16 [9] and the fact $\phi ''\le -\frac{2}{\ell ' r_0}$, we obtain

$$\begin{aligned}&{\varvec{\Delta }} w{|_{X {\setminus } \partial \Omega }}\\&\quad \le -\ell '\phi '(-\ell 'V) {\varvec{\Delta }} V{|_{X {\setminus } \partial \Omega }}-\frac{2\ell '}{ r_0} |\mathrm {D}V|^2\,{\mathfrak {m}}{|_{X {\setminus } \partial \Omega }}\\&\quad \le -\ell '\phi '(-\ell 'V) \Big (\cot _{K, N}({\mathrm {d}}(x, b_q)){\mathfrak {m}}{|_{X {\setminus } \partial \Omega }} +\int _{{\mathfrak {Q}}} h_q \delta _{b_q}\, {\mathrm {d}}{\mathfrak {q}}(q) \Big ) -\frac{2\ell '}{ r_0}\,{\mathfrak {m}}{|_{X {\setminus } \partial \Omega }}. \end{aligned}$$

Furthermore, we know $\phi '(-\ell 'V)=0$ on $\{V \le -\frac{3}{4} r_0\} \cup \{V \ge \frac{3}{4} r_0\}$. So from Lemma 11 we can see that

$$\begin{aligned} \phi '(-\ell 'V) \int _{{\mathfrak {Q}}} h_q \delta _{b_q}=0. \end{aligned}$$

Combining with the monotonicity of $\cot _{K, N}$ we obtain

$$\begin{aligned} {\varvec{\Delta }} w{|_{X {\setminus } \partial \Omega }} \le -\ell ' \Big (\cot _{K, N}( r_0/4) +\frac{2}{ r_0}\Big ){\mathfrak {m}}{|_{X {\setminus } \partial \Omega }} \end{aligned}$$

which is the thesis. $\square$

Combining Theorem 6 and Proposition 5 we can prove the main theorem of this section. Recall that the Minkowski content of a set $Z \subset X$ is defined by

$$\begin{aligned} {\mathfrak {m}}^+(Z):=\mathop {\liminf }_{\epsilon \rightarrow 0} \frac{{\mathfrak {m}}(Z^\epsilon )-{\mathfrak {m}}(Z)}{\epsilon } \end{aligned}$$

where $Z^\epsilon \subset X$ is the $\epsilon$-neighborhood of *Z* defined by $Z^\epsilon :=\{x: {\mathrm {d}}(x, Z)<\epsilon \}$.

#### **Theorem 7**

*Let* $(X,{\mathrm {d}},{\mathfrak {m}})$ *be an* ${\mathrm{{RCD}}}(K, N)$ *space and* $\Omega$ *be a bounded* $\ell$*-convex domain in* $(X, {\mathrm {d}})$ *with* ${\mathfrak {m}}(\partial \Omega )=0$ *and* ${\mathfrak {m}}^+(\partial \Omega )<\infty$. *Then, for any* $N'\in (N, +\infty ]$, *there exists a Lipschitz function* *w* *such that* $({\overline{\Omega }}, {\mathrm {d}}^{w}, {\mathfrak {m}}^w)$ *is a* $\mathrm{RCD}(K', N')$ *space for some* $K' \in {\mathbb {R}}$.

#### *Proof*

Let *w* be the reference function obtained in Proposition 5. Denote by $\mu$ the trivial extension of ${\varvec{\Delta }} w{|_{X {\setminus } \partial \Omega }}$ on whole *X*. To apply Lemma 6 and Proposition 5, it suffices to show that $w \in {\mathrm{{D}}}({\varvec{\Delta }})$ and ${\varvec{\Delta }} w \le \mu$.

Given an arbitrary non-negative Lipschitz function $\varphi \in \mathop {\mathrm{{Lip}}}\nolimits (X, {\mathrm {d}})$ with bounded support. For any $\epsilon >0$, there exists a Lipschitz function $\phi _\epsilon \in \mathop {\mathrm{{Lip}}}\nolimits ({\mathbb {R}})$ satisfying

$$\begin{aligned} \phi _\epsilon (t) :=\left\{ \begin{array}{lll} ~~0, &{}{\text {if}}~~ t\in [0, \frac{\epsilon }{2}]\\ ~~\frac{2}{\epsilon }(t-\frac{\epsilon }{2}), &{}{\text {if}}~~ t\in [\frac{\epsilon }{2}, \epsilon ]\\ ~~1, &{}{\text {if}}~~ t\in [\epsilon , +\infty ) \end{array} \right. \end{aligned}$$

Define ${\bar{\varphi }}_\epsilon := \phi _\epsilon ({\mathrm {d}}(\cdot , \partial \Omega )) \varphi$. By Leibniz rule and chain rule we know $\bar{\varphi }_\epsilon \in \mathop {\mathrm{{Lip}}}\nolimits (X, {\mathrm {d}})$, and $\mathop {\mathrm{{supp}}}\nolimits \bar{\varphi }_\epsilon \subset X {\setminus } \partial \Omega$. Therefore by Proposition 5 and monotone convergence theorem we get

$$\begin{aligned} \int \varphi \, {\mathrm {d}}\mu= & {} \mathop {\lim }_{{\epsilon } \rightarrow {0}} \int {\bar{\varphi }}_\epsilon \, {\mathrm {d}}{\varvec{\Delta }} w{|_{X {\setminus } \partial \Omega }}\\= & {} -\mathop {\lim }_{{\epsilon } \rightarrow {0}} \int _{X {\setminus } \partial \Omega } \Gamma ( {\bar{\varphi }}_\epsilon , w)\, {\mathrm {d}}{\mathfrak {m}}\\= & {} - \mathop {\lim }_{{\epsilon } \rightarrow {0}} \int \phi _\epsilon ({\mathrm {d}}(\cdot , \partial \Omega )) \Gamma (\varphi , w)\, {\mathrm {d}}{\mathfrak {m}}- \mathop {\lim }_{{\epsilon } \rightarrow {0}} \int \varphi \Gamma (\phi _\epsilon ({\mathrm {d}}(\cdot , \partial \Omega )), w)\, {\mathrm {d}}{\mathfrak {m}}\\= & {} - \int \Gamma (\varphi , w)\, {\mathrm {d}}{\mathfrak {m}}- \mathop {\lim }_{{\epsilon } \rightarrow {0}} \int \varphi \Gamma (\phi _\epsilon ({\mathrm {d}}(\cdot , \partial \Omega )), w)\, {\mathrm {d}}{\mathfrak {m}}. \end{aligned}$$

By Theorem 10 we have a measure decomposition ${\mathrm {d}}{\mathfrak {m}}={\mathrm {d}}{\mathfrak {m}}_q \, {\mathrm {d}}{\mathfrak {q}}(q)$ associated with the signed distance function $\pm {\mathrm {d}}(\cdot , \partial \Omega )$. Thus,

$$\begin{aligned}&\mathop {\lim }_{{\epsilon } \rightarrow {0}} \int \varphi \Gamma (\phi _\epsilon ({\mathrm {d}}(\cdot , \partial \Omega )), w)\, {\mathrm {d}}{\mathfrak {m}}\\&\quad = \mathop {\lim }_{{\epsilon } \rightarrow {0}} \int _{\Omega ^{-\epsilon / 2}_{-\epsilon } } \varphi \Gamma (\phi _\epsilon ({\mathrm {d}}(\cdot , \partial \Omega )), w)\, {\mathrm {d}}{\mathfrak {m}}+ \mathop {\lim }_{{\epsilon } \rightarrow {0}} \int _{ \Omega _{ \epsilon /2}^ {\epsilon } } \varphi \Gamma (\phi _\epsilon ({\mathrm {d}}(\cdot , \partial \Omega )), w)\, {\mathrm {d}}{\mathfrak {m}}\\&\quad =\mathop {\lim }_{{\epsilon } \rightarrow {0}} \frac{2\ell '}{\epsilon } \left( \int _{\Omega ^{-\epsilon / 2}_{-\epsilon } } \varphi \, {\mathrm {d}}{\mathfrak {m}}-\int _{ \Omega _{ \epsilon /2}^ {\epsilon } } \varphi \, {\mathrm {d}}{\mathfrak {m}}\right) \\&\quad = \mathop {\lim }_{{\epsilon } \rightarrow {0}} \frac{2\ell '}{\epsilon } \int _{{\mathfrak {Q}}} \Big (\int _{\Omega ^{-\epsilon / 2}_{-\epsilon } \cap X_q} \varphi \, {\mathrm {d}}{\mathfrak {m}}_q-\int _{ \Omega _{ \epsilon /2}^ {\epsilon }\cap X_q } \varphi \, {\mathrm {d}}{\mathfrak {m}}_q \Big ) \, {\mathrm {d}}{\mathfrak {q}}(q). \end{aligned}$$

Notice that

$$\begin{aligned} {\mathfrak {m}}^+(\partial \Omega )=\mathop {\liminf }_{\epsilon \rightarrow 0} \frac{{\mathfrak {m}}\big ((\partial \Omega )^\epsilon \big )}{\epsilon }=\mathop {\liminf }_{\epsilon \rightarrow 0} \frac{1}{\epsilon } \left( \int _{\Omega ^{0}_{-\epsilon } } 1 \, {\mathrm {d}}{\mathfrak {m}}+\int _{ \Omega _{ 0}^ {\epsilon } } 1 \, {\mathrm {d}}{\mathfrak {m}}\right) . \end{aligned}$$

Therefore, we obtain

$$\begin{aligned}&\left| \int \Gamma (\varphi , w)\, {\mathrm {d}}{\mathfrak {m}}\right| \\&\quad \le \left| \int \varphi \, {\mathrm {d}}\mu \right| +\left| \mathop {\lim }_{{\epsilon } \rightarrow {0}} \int \varphi \Gamma (\phi _\epsilon ({\mathrm {d}}(\cdot , \partial \Omega )), w)\, {\mathrm {d}}{\mathfrak {m}}\right| \\&\quad \le \max |\varphi | \left\{ |\mu |(\mathop {\mathrm{{supp}}}\nolimits \varphi )+ \mathop {\lim }_{{\epsilon } \rightarrow {0}} \frac{2|\ell '|}{\epsilon }\left( \int _{\Omega ^{0}_{-\epsilon } } 1 \, {\mathrm {d}}{\mathfrak {m}}+\int _{ \Omega _{ 0}^ {\epsilon } } 1 \, {\mathrm {d}}{\mathfrak {m}}\right) \right\} \\&\quad \le \max |\varphi | \left\{ |\mu |(\mathop {\mathrm{{supp}}}\nolimits \varphi )-2\ell ' {\mathfrak {m}}^+(\partial \Omega )\right\} . \end{aligned}$$

By Riesz–Markov–Kakutani Representation theorem, we know $w \in {\mathrm{{D}}}({\varvec{\Delta }})$.

Since ${\mathfrak {m}}_q=h_q {\mathcal {H}}^1 {|_{X_q}}$ for some ${\mathrm{{CD}}}(K, N)$ probability density $h_q$, we know $h_q$ and $(\ln h_q)'$ are bounded. So for any $X_q$ such that $\Omega ^{- \epsilon /2}_ {-\epsilon }\cap X_q \ne \emptyset$ and $\Omega _{ \epsilon /2}^ {\epsilon }\cap X_q \ne \emptyset$ for $\epsilon$ small enough, we have

$$\begin{aligned} \left| \int _{\Omega ^{-\epsilon / 2}_{-\epsilon } \cap X_q} \varphi \, {\mathrm {d}}{\mathfrak {m}}_q-\int _{ \Omega _{ \epsilon /2}^ {\epsilon }\cap X_q } \varphi \, {\mathrm {d}}{\mathfrak {m}}_q \right|\le & {} \mathop {\mathrm{{Lip}}}\nolimits (\varphi h_q) \frac{3\epsilon }{2} {\mathcal {H}}^1(\Omega _{- \epsilon }^ {\epsilon }\cap X_q)=O(\epsilon ^2). \end{aligned}$$

Hence by Lemma 11 and Fatou’s lemma, we obtain

$$\begin{aligned}&\mathop {\lim }_{{\epsilon } \rightarrow {0}} \frac{1}{\epsilon } \int _{{\mathfrak {Q}}} \Big (\int _{\Omega ^{-\epsilon / 2}_{-\epsilon } \cap X_q} \varphi \, {\mathrm {d}}{\mathfrak {m}}_q-\int _{ \Omega _{ \epsilon /2}^ {\epsilon }\cap X_q } \varphi \, {\mathrm {d}}{\mathfrak {m}}_q \Big ) \, {\mathrm {d}}{\mathfrak {q}}(q)\\&\quad \ge \mathop {\lim }_{{\epsilon } \rightarrow {0}} \frac{1}{\epsilon } \int _{q\in {\mathfrak {Q}}, \Omega ^{r_0}_ {0}\cap X_q \ne \emptyset } \Big (\int _{\Omega ^{-\epsilon / 2}_{-\epsilon } \cap X_q} \varphi \, {\mathrm {d}}{\mathfrak {m}}_q-\int _{ \Omega _{ \epsilon /2}^ {\epsilon }\cap X_q } \varphi \, {\mathrm {d}}{\mathfrak {m}}_q \Big ) \, {\mathrm {d}}{\mathfrak {q}}(q)\\&\quad \ge 0. \end{aligned}$$

In conclusion, we obtain

$$\begin{aligned}&\int \varphi \, {\mathrm {d}}{\varvec{\Delta } } w= -\int \Gamma (\varphi , w)\, {\mathrm {d}}{\mathfrak {m}}\\&\quad = \int \varphi \, {\mathrm {d}}\mu +\mathop {\lim }_{{\epsilon } \rightarrow {0}} \frac{2\ell '}{\epsilon } \int _{{\mathfrak {Q}}} \Big (\int _{\Omega ^{-\epsilon / 2}_{-\epsilon } \cap X_q} \varphi \, {\mathrm {d}}{\mathfrak {m}}_q-\int _{ \Omega _{ \epsilon /2}^ {\epsilon }\cap X_q } \varphi \, {\mathrm {d}}{\mathfrak {m}}_q \Big ) \, {\mathrm {d}}{\mathfrak {q}}(q) \le \int \varphi \, {\mathrm {d}}\mu . \end{aligned}$$

Therefore, ${\varvec{\Delta }} w \le \mu$, by Proposition 5 we know ${\varvec{\Delta }}_\mathrm{{sing}} w \le 0$ and $\big ( \Delta _\mathrm{{ac}} w)^+ \in L^\infty$. Then, by Theorem 6 we know $({\overline{\Omega }}, {\mathrm {d}}^{w}, {\mathfrak {m}}^w)$ is a $\mathrm{RCD}(K', N')$ space. $\square$
